# Temporal and Spatial Variations of Bacterial and Faunal Communities Associated with Deep-Sea Wood Falls

**DOI:** 10.1371/journal.pone.0169906

**Published:** 2017-01-25

**Authors:** Petra Pop Ristova, Christina Bienhold, Frank Wenzhöfer, Pamela E. Rossel, Antje Boetius

**Affiliations:** 1 HGF-MPG Group for Deep Sea Ecology and Technology, Alfred Wegener Institute for Polar and Marine Research, Bremerhaven, Germany, and Max Planck Institute for Marine Microbiology, Bremen, Germany; 2 Research Group for Marine Geochemistry (ICBM-MPI Bridging group), Institute for Chemistry and Biology of the marine environment, University of Oldenburg, ICBM, Oldenburg, Germany; Naturhistoriska riksmuseet, SWEDEN

## Abstract

Sinking of large organic food falls i.e. kelp, wood and whale carcasses to the oligotrophic deep-sea floor promotes the establishment of locally highly productive and diverse ecosystems, often with specifically adapted benthic communities. However, the fragmented spatial distribution and small area poses challenges for the dispersal of their microbial and faunal communities. Our study focused on the temporal dynamics and spatial distributions of sunken wood bacterial communities, which were deployed in the vicinity of different cold seeps in the Eastern Mediterranean and the Norwegian deep-seas. By combining fingerprinting of bacterial communities by ARISA and 454 sequencing with *in situ* and *ex situ* biogeochemical measurements, we show that sunken wood logs have a locally confined long-term impact (> 3y) on the sediment geochemistry and community structure. We confirm previous hypotheses of different successional stages in wood degradation including a sulphophilic one, attracting chemosynthetic fauna from nearby seep systems. Wood experiments deployed at similar water depths (1100–1700 m), but in hydrographically different oceanic regions harbored different wood-boring bivalves, opportunistic faunal communities, and chemosynthetic species. Similarly, bacterial communities on sunken wood logs were more similar within one geographic region than between different seas. Diverse sulphate-reducing bacteria of the Deltaproteobacteria, the sulphide-oxidizing bacteria *Sulfurovum* as well as members of the Acidimicrobiia and Bacteroidia dominated the wood falls in the Eastern Mediterranean, while Alphaproteobacteria and Flavobacteriia colonized the Norwegian Sea wood logs. Fauna and bacterial wood-associated communities changed between 1 to 3 years of immersion, with sulphate-reducers and sulphide-oxidizers increasing in proportion, and putative cellulose degraders decreasing with time. Only 6% of all bacterial genera, comprising the core community, were found at any time on the Eastern Mediterranean sunken wooden logs. This study suggests that biogeography and succession play an important role for the composition of bacteria and fauna of wood-associated communities, and that wood can act as stepping-stones for seep biota.

## Introduction

Sunken wood, kelp and whale carcasses, commonly referred to as large food falls, supply locally large quantities of organic matter to the otherwise food-deprived deep-sea floor [1–4]. These local organic enrichments attract highly adapted and opportunistic fauna and promote the development of prolific ecosystems with one of the highest species richness known from deep-sea habitats [5]. Although individual organic falls affect only localized areas of the deep-sea floor, they occur frequently in all parts of the world’s oceans ([6] and referneces therein). The carbon transported to the deep sea by large organic falls might be negligible compared to the global Particulate Organic Carbon (POC) flux, however, even as one-time events such falls transport substantially higher amounts of carbon than what usually reaches the deep sea floor. For example, recent studies have estimated that a single storm event can transport up to 1.8–4 Tg of driftwood carbon to the ocean, that a single large whale carcass can provide an equivalent of 2000 years of background POC flux to the deep-sea floor, or a sinking swarm of swimming crabs 30–40% of the annual carbon flux [3,7–9]. These estimates clearly indicate the significance of large organic falls for the ecology of deep-sea ecosystems.

The degradation of organic matter derived from large food falls is a temporally dynamic process that involves the succession of specialized communities with distinct life styles and metabolic requirements. Locally, high organic loads may deplete oxygen in the seafloor around the food fall, attracting anaerobic microbial communities to continue degradation via anoxic processes such as fermentation, sulphate reduction and methanogenesis [4,10]. Such anaerobic degradation may alter biogeochemical conditions of the seafloor in the immediate vicinity of such food fall habitats, and cause sulphide production finally attracting chemosynthetic communities [3,4,10–14].

Studies on the temporal succession of food fall communities in the deep sea mostly focused on whale falls as the largest type of carbon input. The degradation of whale carcasses proceeds through four successive stages i.e. 1) mobile-scavenger, 2) enrichment-opportunist, 3) sulphophilic and 4) reef stage, that are distinguished by the fauna colonizing the whale remains and the biogeochemical conditions that evolve [2,3,15]. Specialized macro- and megafauna organisms, e.g. sharks and hagfish initialize the degradation of organic matter from whale carcasses [3]. Their sloppy feeding distributes pieces of meat and fat on the seafloor, and other opportunistic scavengers cause a burial of these food falls into the seafloor [4]. By anaerobic respiration with sulphate, microorganisms produce sulphide and methane, initiating the sulphophilic stage of whale carcasses [2,4,10,16,17], which in turn attracts chemosynthetic organisms commonly found at cold seeps and hydrothermal vents, i.e. symbiotic mytilid mussels, clams, as well as chemoautotrophic bacteria. Based on the commonalities of associated fauna, it has been hypothesized that whale falls and other carcasses might serve as stepping stones in the distribution and evolution of chemosynthetic fauna at seeps and vents [2,18]. A new synthesis study challenges this view and suggests an important role for sedimented vents [19].

Little is known about the temporal succession of communities and biogeochemical gradients at wood falls. Wood-boring bivalves of the family Xylophagaidae are among the first organisms to colonize wood falls in the deep-sea and are responsible for the initialization of the wood degradation by producing wood chips and fecal matter. These provide colonization surfaces and nutrients for other organisms [14,20]. Microorganisms including fungi, play a crucial role in the enzymatic degradation of cellulose [21], one of the major constituents of wood, both under oxic and anoxic conditions [22]. The degradation of cellulose consumes oxygen, with the consequence of anoxic niches developing in and around the wood, especially in nearby sediments where sulphide is produced [14,23]. Similar to whale falls, the following anaerobic degradation by fermenters and sulphate reducing bacteria results in sulphide fluxes as the basis for the establishment of chemoautotrophic communities. Despite their crucial role in the degradation of wood falls, only few studies exist to date focusing on the composition and succession of microbial communities associated to wood falls [14,24–28]. Especially the role of marine fungi remains understudied. Here, the overall aim was to assess dynamics of bacterial communities of wood falls, including temporal and spatial variations.

Therefore, identically designed wood experiments were repeatedly sampled over a time frame of 3 years. The wood experiments were deposited at three geographically distant cold seep sites, located in areas with contrasting environmental conditions, i.e. one in the cold Arctic deep sea (-1°C) and two in the warm, oligotrophic Eastern Mediterranean sea (14°C). Within this study we applied Automated Ribosomal Intergenic Spacer Analysis (ARISA) and 454 Massively Parallel Tag Sequencing (MPTS) to assess temporal and spatial variations in bacterial community structure, combined with *in situ* rate measurements to quantify the influence of wood falls on the biogeochemistry of surrounding sediments. The main questions addressed here are: i) is there a temporal succession of wood-associated bacterial and faunal communities ii) how does the influence of sunken wood on the surrounding sediment bacterial communities and biogeochemistry change with immersion time, iii) can we identify geographical patterns in the distribution of wood-associated bacterial and faunal communities, and iv) are there core wood-associated bacterial communities across oceans.

## Material and Methods

### Description and sampling of sunken wood experiments

**Fig 1:**
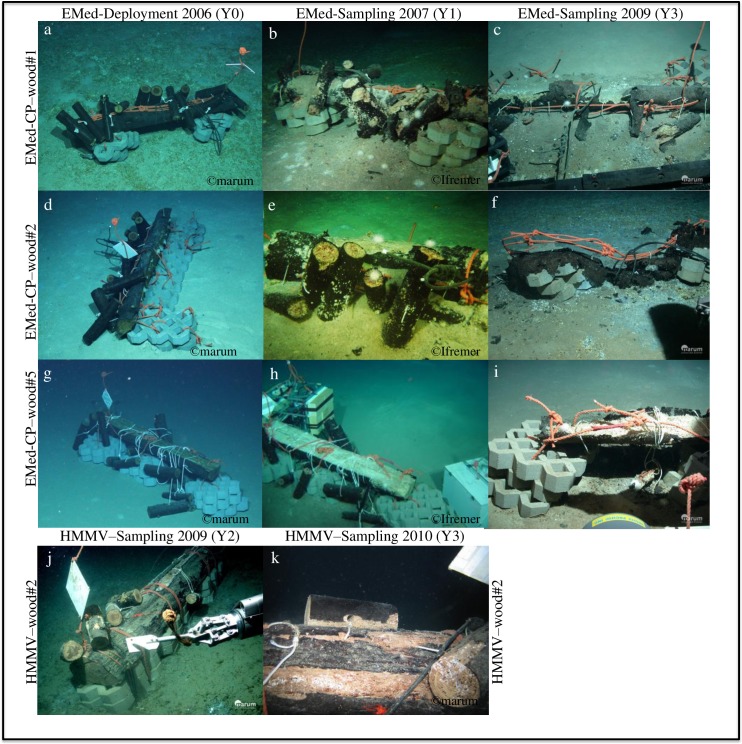
ROV-based images of the wood experiments. Photos portray the condition of the wood experiments during the deployment and sample recovery in the (a–i) Eastern Mediterranean sea (Central Province) and the (j–k) Norwegian sea (HMMV). Pictures a, c, d, f, g, i, j, k reprinted with permission from MARUM, University Bremen, Germany. Pictures b, e, h reprinted from [14] under a CC BY license, with permission from PLOS ONE, original copyright 2013.

A batch of wood logs from Douglas fir (*Pseudotsuga menziesii*), standardized in size and age (trees of 30 y), were obtained from forestal land in Germany for a consistent set-up of the experiments. This type of wood was chosen to be consistent with earlier wood fall experiments [6,21,29]. The wood logs were stored in a container for roughly 6 months before deployment. At the time of deployment there were no apparent signs of degradation by fungi or bacteria (e.g. no molds, blackening or biofilms had developed). Each wood colonization experiment consisted of one large wood log (200 x 30 cm) of Douglas fir, to attract large fauna colonizers, and several small attached wood logs (30–50 x 10–15 cm) that could be repeatedly sampled (Fig 1) [14]. Cement blocks were attached as weights. In total, nine wood experiments (Table 1) were deployed at three cold seep sites i.e. the Central Province (CP) of the Nile Deep-Sea Fan characterized by carbonates and pockmarks and the Amon Mud Volcano (AMV) in the Eastern Mediterranean Sea (EMed) [30–33], and the Håkon Mosby Mud Volcano (HMMV) in the Norwegian Sea (NorS) [34,35]. The most prominent difference between these cold seep sites besides their geographic location, is the bottom-water temperature, which for the Norwegian Sea is– 1°C, and for the two deep Eastern Mediterranean sites 14°C.

**Table 1:** Location of wood experiments in the Norwegian and the Eastern Mediterranean deep sea, description of the benthic habitats and date of deployment and samplings events.

Wood experiment	Location	Habitat	Position	Deployment; Cruise	Sampling; Cruise
**EMed-CP-wood#1**	Eastern Mediterranean; Central Province	On sediments within seep area; close to carbonate crusts	N 32°32.0496	Nov 2006; Bionil^1)^	Nov 2007; Medeco^2)^
			E 30°21.1248		Oct-Nov 2009; MSM13^3)^
**EMed-CP-wood#2**	Eastern Mediterranean; Central Province	On carbonate crust within seep area	N 32°31.9626	Nov 2006; Bionil^1)^	Nov 2007; Medeco^2)^
			E 30°21.1752		Oct 2009; MSM13^3)^
**EMed-AMV-wood#3**	Eastern Mediterranean; Amon Mud Volcano	On sediments within seep area	N 32°21.9920	Nov 2006; Bionil^1)^	Nov 2009; MSM13^3)^
			E 31°42.1788		
**EMed-AMV-wood#4**	Eastern Mediterranean; Amon Mud Volcano	On sediments within seep area	N 32°22.0159	Nov 2006; Bionil^1)^	Nov 2009; MSM13^3)^
			E 31°42.2205		
**EMed-CP-wood#5**	Eastern Mediterranean; Central Province	On sediments outside seep area	N 32°32.0790	Nov 2006; Bionil^1)^	Nov 2007; Medeco^2)^
			E 30°21.3840		Nov 2009; MSM13^3)^
**EMed-CP-wood#6**	Eastern Mediterranean; Central Province	On sediments within seep area; close to carbonate crusts	N 32°32.0124	Nov 2007; Medeco^2)^	Nov 2007; Medeco^2)^
			E 30°21.1920		Nov 2009; MSM13^3)^
**EMed-CP-wood#7**	Eastern Mediterranean; Central Province	On sediments within seep area	N 32°32.0775	Nov 2009; MSM13^3)^	Nov 2009; MSM13^3)^
			E 30°21.3862		
**NorS-HMMV-wood#1**	Norwegian Sea; Håkon Mosby Mud Volcano	On sediments within seep area	N 72° 00.3900	Jun 2007; ARKXXII/1b^4)^	Jul 2009; ARKXXIV/2^4)^
			E 14° 43.6398		Oct 2010; MSM16^5)^
**NorS-HMMV-wood#2**	Norwegian Sea; Håkon Mosby Mud Volcano	On sediments within seep area	N 72° 00.3780	Jun 2007; ARKXXII/1b^4)^	Jul 2009; ARKXXIV/2^4)^
			E 14° 43.0957		
**Wood tiles in nets experiment**	Norwegian Sea; Håkon Mosby Mud Volcano	On sediments within seep area	N 72° 00.3780	Jul 2009; ARKXXIV/2^4)^	Oct 2010; MSM16^5)^
			E 14° 43.0957		

1) RV Meteor; ROV Quest 4000

2) RV Pourquoi Pas?; ROV Victor 6000

3) RV Maria S Merian; ROV Quest 4000

4) RV Polarstern; ROV Quest 4000

5) RV Maria S Merian; ROV Genesis.

The Central Province area, at a water depth of around 1700 m, encompasses numerous small pockmarks (circular depression few meters in diameter and approximately one meter deep) associated with active seepage of methane, which in turn supports the development of bacterial mats and precipitation of large flat authigenic carbonate crusts that overlie reduced sediments [30,34,36]. The wood experiments in the Central Province were deployed in different habitats: on deep-sea muds (EMed-CP-wood#5, EMed-CP-wood#7), on carbonate crust (EMed-CP-wood#2) and close to carbonate crust (EMed-CP-wood#1, EMed-CP-wood#6) (Table 1, see also [14]). Wood samples were obtained from the experiment in the Central Province deployed in 2006 after one year (in 2007; EMed-CP-wood#1-Y1, EMed-CP-wood#2-Y1, EMed-CP-wood#5-Y1) and three years of immersion (in 2009; EMed-CP-wood#1-Y3, EMed-CP-wood#2-Y3, EMed-CP-wood#5-Y3) (Table 1; Fig 1A–1I). EMed-CP-wood#6 was deployed in 2007 and sampled after 1 day of deployment (EMed-CP-wood#6-Y0) to serve as a starting point, at which no significant colonization could have taken place; it was also sampled two years later in 2009 (EMed-CP-wood#6-Y2). A detailed description of the sampling procedure and biogeochemical measurements performed on these wood experiments after one year of immersion, in 2007, is given in [14]. In 2009 an additional wood experiment EMed-CP-wood#7 was deployed in the vicinity of the EMed-CP-wood#5, to serve as a reference for the investigation of the impact of wood on the surrounding sediments (Table 1).

At the Amon Mud Volcano (Ø 2 km, water depth 1120 m), two wood experiments were deployed in 2006 on sediments close to a sulphidic mud flow at the foot of the mud volcano [31,37,38]. Both experiments were for the first time sampled after three years of submersion (EMed-AMV-wood#3-Y3 and EMed-AMV-wood#4-Y3; Table 1).

At the Håkon Mosby Mud Volcano in the Norwegian Sea (Ø 1.4 km, water depth 1250 m) two wood experiments (NorS-HMMV-wood#1 and NorS-HMMV-wood#2) were deposited in 2007 on a so-called “siboglinid tubeworm” habitat (Table 1) [39–41]. This habitat was characterized by sediments densely populated with two symbiotic species of siboglinid annelids i.e. *Sclerolinum contortum* and *Oligobrachia haakonmosbiensis* [42,43]. Samples from NorS-HMMV-wood#1 were obtained after two (in 2009; NorS-HMMV-wood#1-Y2) and three years (in 2010; NorS-HMMV-wood#1-Y3) of submergence (Fig 1J and 1k). Due to limited ROV dive time, samples from NorS-HMMV-wood#2 were only obtained in 2009 (NorS-HMMV-wood#2-Y2). In addition, we tested if wood-boring bivalves are able to settle on freshly submerged wood tiles when these are wrapped in nets of different mesh sizes (9 mm, 0.33 mm and 0.055 mm). The nets with wood tiles (nine in each net) were incubated for 1 year attached to a bottom lander system at the same location (HMMV at 1250 m water depth) (S1 Fig).

A summary of the location, type of habitat, deployment and sampling events of the different wood experiments is given in Table 1. All metadata are stored in the PANGAEA database (http://www.pangaea.de/), and PANGAEA references for the samples are cited accordingly (S1 Table; [14]).

### Visual observations and sampling of wood experiments

ROV-based high quality video and photo surveys were performed on the wood experiments prior to sampling, in order to assess the condition of the sunken wood logs, to qualitatively deduce potential changes in the degradation rates with increasing time and to observe associated megafauna. Before sampling of the wood logs, we took note of the fauna associated with the logs, and visually inspected the logs for epi- and infauna upon onboard retrieval and before taking the microbiological samples. We used the species catalogue from [14] to identify the fauna associated with wooden logs in the Eastern Mediterranean, and [29,42] for those from Norwegian Sea. Quantitative retrieval of wood logs and fauna from the deep-sea floor was not possible with the ROV due to the state of degradation of the logs after 1–3 years.

Wood log samples were recovered from the Norwegian and Eastern Mediterranean experiments, and subsampled for bacterial community analyses, as well as assessment of macro-, and mega-faunal colonizers. The *in situ* and on board subsampling procedures of the wood logs were conducted as described in [14]. From each wood experiment three small wood logs were sampled at each sampling time point. After 3 years, the degradation of some of the wood logs had advanced substantially, thus in some cases it was not possible to recover three complete wood logs per experiment. Moreover, some of the wood logs were so heavily degraded that it was not possible to distinguish the surface from the subsurface. However, when possible, 2 x 3 subsamples of the surface (0–2 cm) and the subsurface (2–4 cm), as well as an additional sample from the bark, were obtained from each wood log, resulting in maximum 21 samples per wood experiment. Wood samples were stored at -20°C for DNA analyses.

For most of the Eastern Mediterranean experiments additional sampling was performed of adjacent wood-influenced sediments labeled as “At-wood” (e.g. EMed-CP-At-wood#1-Y3; max. distance to wood 0.5 m;) and reference sediments, labeled as “Away-wood” (e.g. EMed-CP-Away-wood#5-Y3; max. distance to wood 10 m). This served to determine the degradation effects of the wood on the sediment bacterial communities and the biogeochemistry. Biogeochemical measurements and sediment retrieval was not possible at the EMed-CP-wood#2, as the wood logs were deposited on top of hard carbonate substrate. Due to limitation in ROV-bottom time, no *in situ* biogeochemical analyses of wood-influenced sediments could be performed for the Norwegian Sea wood experiments. Sediments were sampled with the help of ROVs using push core liners (Ø 8 cm, 20–30 cm sediment depth), or ship-based Multiple Corer (MUC; Ø 9.5 cm). On board the cores were sliced in 1 cm resolution and subsampled for DNA analyses.

### Microbial community analysis

#### DNA extractions

Prior to DNA extraction, wood samples were manually cut under aseptic conditions into small wood chips of < 3 mm size using a sterile scalpel. Total DNA was extracted from finely cut wood material (0.3 g) or sediment (1 g) using UltraClean Soil DNA Isolation Kits (MoBio Laboratories Inc., Carlsbad, CA). DNA was finally eluted in 50 μl 1 x Tris-EDTA buffer (Promega, Madison, WI, USA), and its concentration was determined with a microplate spectrometer (Infinite® 200 PRO NanoQuant, TECAN Ltd, Switzerland).

#### Automated ribosomal intergenic spacer analysis (ARISA)

ARISA amplification [44] of bacterial DNA in triplicates was performed using peqGOLD Taq Polymearase (PEQLAB Biotechnologie GMBH, Erlangen, Germany) and the ITSR and Hex-labeled ITSF primers [45]. Subsequent capillary electrophoresis analyses and *in silico* binning (using a bin size of 2 bp; Interactive Binner function, http://www.mpi-bremen.de/en/Software_4.html.) was carried out according to [14]. Briefly, fragments between 100 and 1000 base pairs were binned into operational taxonomic units (OTUs). The three ARISA PCR replicates were merged to form a consensus profile under stringent conditions, where a peak was considered present if it appeared in at least two out of the three PCR replicates.

#### 454 Massively parallel tag sequencing

Using the same DNA extracts, the bacterial communities of wood and sediment samples were also sequenced with 454 MPTS technology. For this purpose, one sample of the wood subsurface was selected from each wood experiment and from all immersion periods. The V4 –V6 hypervariable region of the 16S rRNA gene was sequenced by pyrosequencing on a Genome Sequencer FLX system (Roche, Basel, Switzerland) at the Marine Biological Laboratory in Woods Hole, MA, USA (for detailed information see http://vamps.mbl.edu), using the 518F and 1064R primers. Samples were amplified following a standardized protocol by mixing 1X Platinum HiFi *Taq* polymerase buffer, 1.6 units Platinum HiFi polymerase (Life Technologies, Carlsbad CA), 3.7 mM MgSO_4_, 200 μM dNTPs (PurePeak polymerization mix, ThermoFisher, E. Providence RI), and 50 nM combined primers, as described in [46]. DNA of 5–25 ng was added to the master mix (final volume of 100 μl), which was then divided in three replicates of 33 μl. Amplification was performed on a Applied Biosystems 2720 or 9700 cycler (Life Technologies) including the following steps: initial denaturation at 94°C for 3 minutes, 30 cycles of 94°C for 30 seconds, 60°C for 45 seconds and 72°C for 1 minute, and a final extension at 72°C for 2 minutes. Raw sff files, upstream data processing and taxonomy assignment was done with the *mothur* (v.1.29; [47]) and the SILVAngs analysis pipeline [48], as described elsewhere [49]. Operational Taxonomic Units (OTU_0.03_) were formed by clustering the sequences at 97% similarity cut-off value. All analyses were performed excluding singletons, i.e. OTU_0.03_ represented by only one sequence in the whole dataset. Characteristics of the 454 MPTS dataset are given in S2 Table. SFF files of all sequence data are publically available at the GenBank Sequence Read Archives (http://www.ncbi.nlm.nih.gov) under BioProject ID: SRP084215.

Analyses of temporal patterns based on the samples retrieved in 2007 (V6-based sequences, provided by [14] and 2009 (V4-V6-based sequences, this study) from the Eastern Mediterranean, were based on only the V6 hypervariable region. For this purpose the V6 hypervariable region was excised from the V4-V6-based dataset and datasets were processed via *mothur* as described in [49].

#### Statistical analyses

Beta-diversity patterns of the bacterial community structure and composition were visualized by Non-metric MultiDimensional Scaling (NMDS) based on ARISA OTU tables. For this analysis the Bray-Curtis distance index was used, as this index is suited for datasets with many double-absence occurrences [50]. Analysis of similarity (ANOSIM) was used to test for significant differences between *a posteriori* groupings of samples. To assess spatial patterns in the bacterial community structure, samples submerged for the same period of time i.e. 3 years were compared. Spearman correlation between bacterial community differences and geographic distance were tested with the Mantel test using 999 Monte-Carlo permutations, and resulting mantel p-values were corrected for multiple testing using the Bonferroni’s correction [51]. Samples originating from the same wood experiments, but sampled at different time points were used to assess the temporal succession of bacterial communities. All statistical analyses were performed in R (v. 2.9.1) (R Development Core Team 2009, http://www.R-project.org) using *vegan* [52] and custom R scripts.

### Biogeochemical analysis

#### Sediment porewater analyses

Pore water extraction using Rhizon moisture samplers [53] were performed at centimetre resolution, from 0.5 cm to 20 cm. Subsamples (1 ml) for sulphate and sulphide analyses were fixed with 0.5 ml of zinc acetate and stored at 4°C until further processing. Total sulphide concentrations (H_2_S + HS^-^ + S_2_^-^) were determined with the diamine complexation method [54].

Additional porewater samples were collected for the analyses of dissolved organic matter (DOM) from EMed-CP-wood#1-Y1, EMed-CP-wood#5-Y1 and their respective reference sediments after 1 year of immersion. Here, porewater was extracted by centrifugation (3500 x g, 10 Min) and filtered through 0.22 μm cellulose-acetate filters. Measurements of DOC and TDN, as well as DOC fluxes in the sediment are presented elsewhere [14]. Porewater samples were solid phase extracted (SPE) using 100 mg styrene divinyl benzene polymer column (Varian PPL) prior to mass spectrometry [55]. To chemically identify the components of wood at the level of the molecular formulae, DOM molecular analysis was performed with a Solarix 15 Tesla FT-ICR-MS instrument (Bruker Daltonic) using electrospray ionization (ESI; Apollo II Bruker Daltonik GmbH, Bremen, Germany) in negative ion mode (Research Group for Marine Geochemistry, University of Oldenburg). Molecular formula assignments were based on previously reported criteria [56]. See supplement text for further detailed information.

#### *In situ* quantification of total oxygen uptake (TOU)

The total oxygen uptake of wood-influenced sediments (EMed-AMV-At-wood#4-Y3 and EMed-AMV-At-wood#6-Y3) and reference sediments (EMed-AMV-Away-wood#4-Y3 and EMed-AMV-Away-wood#6-Y2) was determined *in situ* with a benthic chamber module [35,57] (S1 Table). The centrally stirred ROV-benthic chamber module (CHAM) enclosed 284 cm^2^ (Ø 19 cm) of sediment surface with 10–25 cm of overlying bottom water (equivalent to 4–6 L). Once at the seafloor, the benthic chamber was handled and operated by a ROV, which ensured precise positioning of the chamber module to the sites of interest. Upon deployment of the chamber to the seafloor, a one-way valve released the excess water and thus provided smooth placement on the sediment. The exact height of the enclosed water body was determined with the help of the ROV-camera system. The decline in bottom water oxygen concentration during the incubation was continuously measured with an oxygen optode (AADI, Norway). In general, the initial decrease in oxygen concentration with time was used to calculate the TOU (mmol m^-2^ d^-1^), as given in Felden et al. (2010):

$$TOU=\frac{dC}{dt}\;*\;\frac{Vchamber}{Achamber}$$

where, dC/dt (μmol L^-1^ h-1) is the change of oxygen concentration over incubation time, V_chamber_ (cm^3^) is the volume of the overlying water in the enclosed chamber, and the A_chamber_ (cm^2^) is the area of the sediment enclosed by the chamber.

#### *In situ* and *ex situ* quantification of diffusive oxygen uptake (DOU)

Diffusive oxygen uptake and Oxygen Penetration Depth (OPD) in sediments at (0.5 m) and away (10 m) from the wood experiments were determined via microsensor measurements, performed both *in situ* using a ROV-microprofiler module (MICP) and *ex situ* on retrieved push cores (for details see [58]. A list of all microsensor measurements performed in 2009 is given in the supplementary S1 Table. *Ex situ* microsensor measurements (including O_2_, H_2_S and pH profiles) performed at the wood experiments in 2007 are available in [14]. Calibration of the O_2_-microsensors and measurement of *in situ* high-resolution (200 μm) O_2_ concentration profiles was performed as described elsewhere [4,59–61]. The laboratory set up used to conduct *ex situ* oxygen microsensor measurements was done according to [14]. Based on the Fick’s first law of diffusion, the *in situ* and *ex situ* DOU was calculated from the linear oxygen concentration gradient in the diffusive boundary layer (DBL), according the formula [62]:

$$J=D\;*\;\frac{\delta C}{\delta z}$$

where J is the diffusive flux in mmol m^-2^ d^-1^, D is the diffusion coefficient of oxygen in water (D = 1.6 x 10^−9^ m^2^ s^-1^), corrected for the temperature and salinity [63] and δC/δz is the concentration gradient (mmol m^-2^) through the DBL.

#### *Ex situ* measurements of sulphate reduction (SR) and methane consumption (MOx)

At each of the investigated sites (S1 Table), three sediment replicate subcores (Ø 2.8 cm) were injected at 1 cm resolution with either 25 μl ^14^CH_4_ (dissolved in water, 2,5 kBq) or 5–10 μl ^35^SO_4_^-2^ (dissolved in water, 50 kBq) for the quantification of Sulphate Reduction (SR) and Methane Oxidation (MOx) rates, according to the whole-core injection method [64]. The injected subcores were incubated for approx. 12 h at an *in situ* temperature of 14°C in the dark. Afterwards, the cores were sliced at 1 cm resolution and fixed with NaOH (2.5%, w/v, in glass gas tight bottles) or 20 ml zincacetate (20%, w/v) for MOx and SR analyses, respectively. Substrate (methane and sulphate) concentrations were measured by gas chromatography (5890A Hewlett Packard) and anion exchange chromatography (Waters IC-Pak anion exchange column, waters 430 conductivity detector), respectively. Turnover rates were measured in the home laboratory and calculated according to [65,66].

Diplomatic permits were obtained from Norwegian and Egyptian authorities to work in waters under Norwegian and Egyptian jurisdiction. No specific permits were required for the field study in the Central Eastern Mediterranean, which was in international waters. Douglas fir trees were obtained from a local forest manager in Germany, permitted to cut wood. The field studies did not involve endangered or protected species.

## Results

### *In situ* visual observations

The sunken wood experiments deposited in the Central Province area and the Amon Mud Volcano (Eastern Mediterranean) showed a progressing degradation by wood-boring bivalves after 1 (2007) and 3 (2009) years of submergence (Fig 1A–1I). Big piles of wood chips of 1–5 cm height, produced by the activity of the wood-boring bivalves, were spread over the sediments surrounding all experiments within a distance of up to 0.5 m around the central log (areal coverage of approximately 4.5 m^2^). At the Central Province, the small wood logs were increasingly degraded with time over the 3 years, and only parts of the bark of the large wood logs were still present. In contrast, at Amon Mud Volcano, after 3 years the sunken wood logs still contained most of the originally deployed material, with most of the small logs still attached to a more or less complete large wood log. Blackened, reduced spots, indicating precipitation of iron sulphide, existed within the woodchip accumulations and at the interface to the underlying sediments. The extent of these reduced spots was most pronounced at EMed-CP-wood#6-Y2, encompassing the surrounding sediment up to a distance of 0.5–1 m. White debris, most probably derived from broken shells of woodborers, was sprinkled around the wood experiments. White sea urchins, amphipods, large crustaceans and occasionally snails were the most prominent fauna dwelling on the wood chips and the sunken wood logs in the Eastern Mediterranean (Table 2).

**Table 2:** List of the most prominent and abundant fauna colonizing the wood experiments in the Norwegian and Eastern Mediterranean Sea.

	EMed-CP-wood#1-Y1	EMed-CP-wood#1-Y3	EMed-CP-wood#2-Y1	EMed-CP-wood#2-Y3	EMed-CP-wood#5-Y1	EMed-CP-wood#5-Y3	EMed-AMV-wood#3-Y3	EMed-AMV-wood#4-Y3	EMed-CP-wood#6-Y2	NorS-HMMV-wood#1-Y2	NorS-HMMV-wood#1-Y3	NorS-HMMV-wood#2-Y2
Sipunculids* (*Phascolosoma turnerae*)	+	+	+	+	+	+	+	+	+	-	-	-
Amphinomid*	+	+	+	+	+	+	+	-	+	-	-	-
*Xylophaga dorsalis*	+	+	+	+	+	-	-	-	-	-	-	-
*Xylophaga brava*	-	+	-	+	-	+	+	+	+	-	-	-
*Xyloredo*	-	-	-	-	-	-	-	-	-	+	+	+
*Idas modiolaeformis*	+	+	+	+	+	+	(+)	(+)	+	-	-	-
Galatheidae	-	+	-	+	-	+	-	-	-	-	-	-
Amphipod	+	-	+	-	+	-	+	+	-	-	-	+
Gastropod	-	+	-	+	-	-	-	-	-	-	-	-
Sea urchin* (*Asterechinus elegans*)	+	+	+	+	+	+	-	+	+	-	-	-
*Glycera noelae sp*. *nov*.	+	-	+	-	+	+	-	-	-	-	-	-
Siboglinidae	-	-	-	-	-	-	-	-	+	+	+	+
Pycnogonids	-	-	-	-	-	-	-	-	+	+	-	-
									

* Fauna sampled in 2007 has been taxonomically identified to species level (see Bienhold et al., 2013).

The degree of degradation of the Norwegian Sea wood experiments appeared more variable among different wood experiments and among wood logs within individual experiments (Fig 1J and 1K). Both sunken wood experiments were deployed on deep-sea muds densely populated by chemosynthetic siboglinid tubeworms. In the case of NorS-HMMV-wood#1-Y2, after 2 years of submergence, the sediments directly surrounding and up to a distance of 0.5 m of the wood experiments were covered with white bacterial mats, resembling the thiotrophic mats of active seeps around HMMV [41]. The most striking difference to the Eastern Mediterranean sunken wood experiments was the lack of wood chips. The only larger fauna residing on the sunken wood logs were few crustaceans and pycnogonids. The wood tiles incubated in nets attached to a lander at HMMV (Norwegian Sea) showed the beginning degradation of the wood by wood-boring bivalves after 1 year (S1 Fig). The 9 mm net-tiles showed most borings (71 ± 35, n = 9), less were present in the 0.33 mm net (27 ± 28, n = 9) and no borings were found in the 0.05 mm net, indicating that the bivalves probably disperse and settle on wood in the larval stage at sizes >0.3 mm (S1 Fig).

### Wood-associated faunal communities

The faunal communities associated with wood falls in the Eastern Mediterranean changed visibly within the 3 years since the deployment of the experiments. We saw fewer living wood-boring bivalves, but more sipunculids upon visual inspection (Table 2). In addition to *Xylophaga dorsalis* already recovered in 2007 and making up most of the shell littering around the wooden logs, a second species from the subfamily Xylophagainae, namely *Xylophaga brava* [67] was recovered from the sunken wood experiments in 2009. Chemosynthetic bivalves present on the wood experiments in 2007 and 2009 in the Central Province and also at Amon Mud Volcano were identified as *Idas modiolaeformis* [68], which are known to populate the nearby seeps (Table 2) [14,33]. Sea urchins (*Asterechinus elegans*), swarms of small amphipods and some crabs were the largest fauna colonizing the experiments and also the piles of wood chips covering the sediments. Up to 4–5 sea urchins were observed per experiment, amounting to only one fifth of the number of specimens encountered 2y before [14].

**Fig 2:**
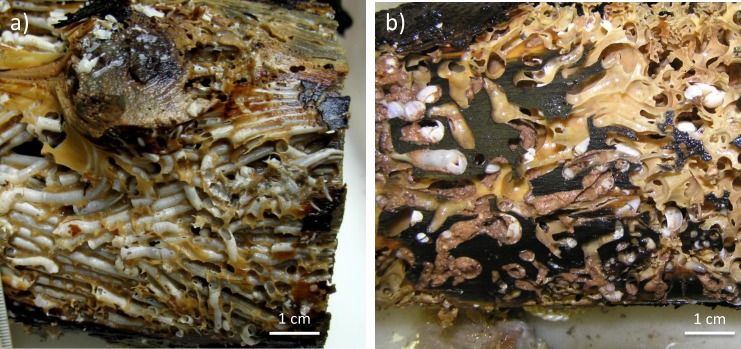
A close-up of wood logs colonized by wood-boring bivalves. Photos depict prominent differences in the degradation of wood experiments by wood-boring bivalves in the Norwegian (a) and Eastern Mediterranean Sea (b). Burrows with calcareous linings built by *Xyloredo ingolfia* at the Norwegian Sea wood experiment are visible on the left panel (a). The right panel shows the burrows of *Xylophaga* spp., which produce large quantities of wood chips (see Fig 1).

At the wood falls deployed at HMMV in the Norwegian Sea, the wood-boring bivalves belonged to *Xyloredo ingolfia* (J. Voight, C. Borowski pers. comm) known from the northern Atlantic. This *Xyloredo* makes burrows with white calcareous linings, which stretched longitudinally throughout the complete wood logs and occupied extensive parts of the logs. In some cases the linings completely substituted the typical structure of the wood logs (Fig 2). The chemosynthetic siboglinid tubeworm *Sclerolium contortum* (Gaudron S., Université Pierre et Marie Curie, pers. communication) was found at the outer side of the sunken wood logs in 2009 and 2010 (Table 2). In contrast to the Eastern Mediterranean experiments, pycnogonids were the dominant predatory animals associated to the Norwegian Sea wood logs. No sea urchins were observed on the wood, but also never in the background HMMV area [42].

### Bacterial communities of wood-associated (wood falls) and wood-influenced sediments (wood-chip)

#### Wood-associated bacterial communities

**Fig 3:**
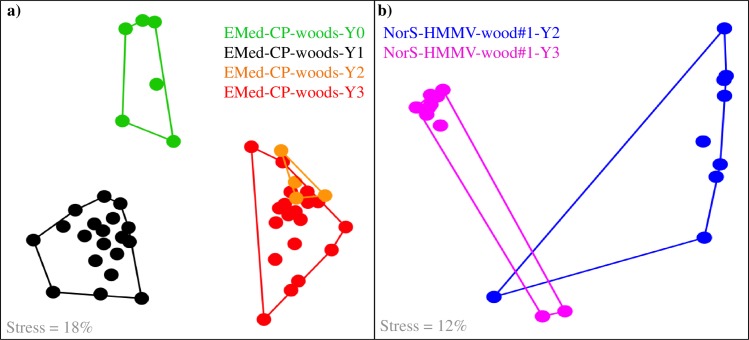
NMDS analysis revealing temporal variations in the bacterial community structure, based on ARISA. The Eastern Mediterranean Sea NMDS analysis (a) includes samples derived from EMed-CP-wood#1, EMed-CP-wood#2, EMed-CP-wood#5, immersed for one and three years, EMed-CP-wood#6 submerged for one day and two years, and EMed-CP-wood#7 deployed for one day in the Central Province. The Norwegian Sea NMDS analysis (b) includes samples from NorS-HMMV-wood#1 immersed for two and three years. Samples are color coded according to immersion time. Convex hulls depict significant differences between the groups, as determined by the analysis of similarity (ANOSIM R = 1, Bonferroni corrected p < 0.01, see also S4 Table).

Temporal variations in the wood-associated bacterial community structures were observed in all experiments. Shifts in the wood bacterial community structures occurred between 1d, 1y, 2y and 3y (EMed-CP-wood#1, EMed-CP-wood#2, EMed-CP-wood#5, EMed-CP-wood#6, NorS-HMMV-wood#1), as revealed by the NMDS analysis (Fig 3). They also occurred in the wood tiles protected by nets and attached to a lander 1.5 m above bottom, where the community changed within 1 y, linked to the emergence of wood boring bivalves (S1 Fig). An ANOSIM test based on the ARISA data showed significant temporal variations in the Eastern Mediterranean Sea and to certain extent also in the Norwegian Sea (S4 Table).

**Fig 4:**
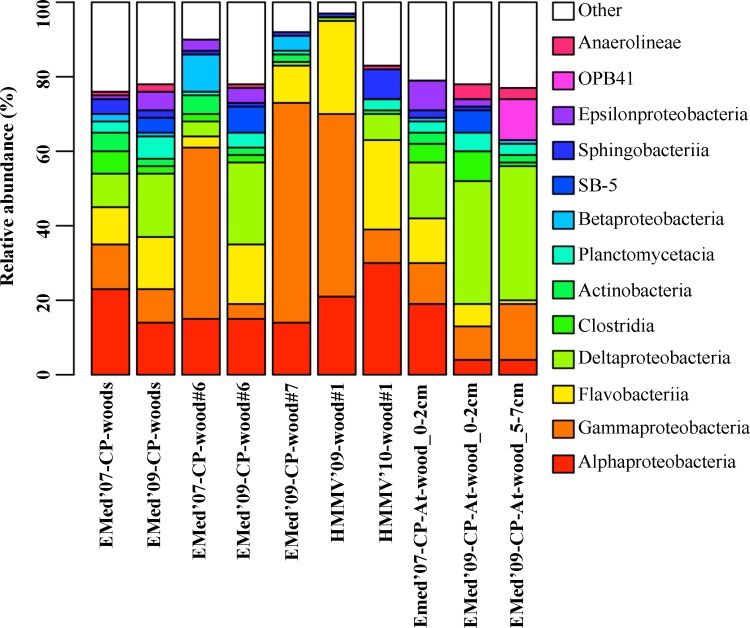
Temporal variation in the taxonomical composition of bacterial classes during three years of submergence. For the first two bars, data from all wood experiments (EMed-CP-wood#1, EMed-CP-wood#2 and EMed-CP-wood#5) of the Central Province in the Eastern Mediterranean immersed for one (Y1) and three (Y3) years, respectively, were pooled together.

Different taxa responded differently to the increasing period of immersion. Of the most dominant bacterial classes, the relative abundance of Alphaproteobacteria and Clostridia decreased, while that of Delta- and Epsilonproteobacteria increased with immersion time at all wood logs deployed in the Central Province (Fig 4, S5 Table). At the genus level, *Pir4* lineage (Planctomyceta), *Sulfurovum* (Epsilonproteobacteria), *Desulfobulbus* and SEEP-SRB4 of Deltaproteobacteria increased with time, while *Tenacibaculum*, *Maribacter* and *Leadbetterella* of the Bacteroidetes decreased with time (S6A Table). Only 22 out of the total 376 (6%) genera were present during the whole submergence period of the wood experiments (1y, 2y and 3y submerged) in the Central Province, comprising 33% of all OTU_0.03_ sequences. Of these, *Pir4* lineage (7%), *Demequina* (5%), *Sulfurovum* (3%), *Sulfurimonas* (2%), *Leisingera* (2%) and *Desulfobulbus* (4%), *Desulfobacula* (4%) and SEEP-SRB4 (5%) were the most sequence abundant ones.

Temporal differences at the class level were even more prominent at the NorS-HMMV-wood#1 deployed in the Norwegian Sea for 2 and 3 years. Alphaproteobacteria, Sphingobacteriia and Deltaproteobacteria increased in relative sequence abundance with immersion time (Fig 4). Conversely, a substantial decrease in the relative abundances was observed for Gammaproteobacteria, while Flavobacteriia remained the same during the immersion period. *Pseudahrensia* (18%; Alphaproteobacteria), *Tenacibaculum* (10%), *Maribacter* (9%) were the most abundant among the 28 common genera (24% of the 118 in total) found during the whole immersion period of 3 years (S6B Table). Overall the common genera comprised 62% of the total OTU_0.03_ sequences.

**Fig 5:**
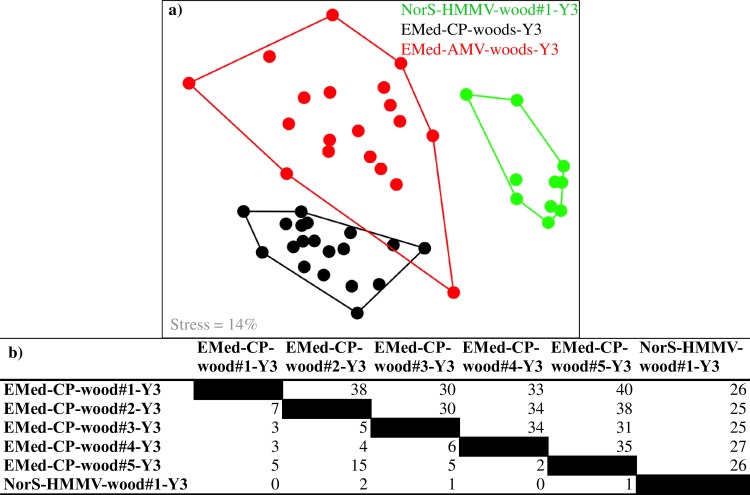
Spatial variations in the wood-associated bacterial community structure. 3D NMDS analysis (Bray-Curtis dissimilarity) (a) and percentage of shared 454 OTU_0.03_ (lower triangle) and ARISA OTUs (upper triangle) (b), depicting spatial variations in the structure of wood bacterial communities (immersed for 3 years) between wood experiments of different geographic regions i.e. Central Province, Amon Mud Volcano and the Håkon Mosby Mud Volcano. ARISA data used for the NMDS analysis includes samples of the following wood experiments: EMed-CP-wood#1-Y3, EMed-CP-wood#2-Y3, EMed-CP-wood#5-Y3, EMed-AMV-wood#3-Y3, EMed-AMV-wood#4-Y3 and the NorS-HMMV-wood#1-Y3). The complete 3D NMDS configuration is displayed in S4 Fig.

To investigate spatial variations in wood-associated bacterial communities, experiments submerged for the same period of time (3y), but deployed at different sites were compared. Pairwise comparison showed that highest percentage of shared OTU_0.03_ (8 ± 5% SD, n = 5) was found between wood experiments of the same locality, less between wood logs within the wider region (4 ± 1% SD, n = 5), and least between logs of the two different seas (1 ± 1% SD, n = 6) (Fig 5B). Bacterial community structures on wood experiments deposited in the Eastern Mediterranean or Norwegian Sea were significantly separated, but with some overlap (ANOSIM R = 0.4–0.6, Bonferroni corrected p-value = 0.001; Fig 5, S7 Table) [69], based on the ARISA dataset. In contrast, bacterial communities of wood experiments located within the same locality had highly overlapping structure (Fig 5 and S4 Fig).

Only 13 out of 310 genera (20% of the total number of sequences) were common to all 3y-immersed wood logs, of which *Pir4 lineage* (5%), *Sulfurovum* (4%), *Demequina* (3%), *Rhodobium* (2%, Alphaproteobacteria) and *Pseudospirillum* (1%; Gammaproteobacteria) were the most abundant. No single OTU_0.03_ was found at all wood experiments.

**Fig 6:**
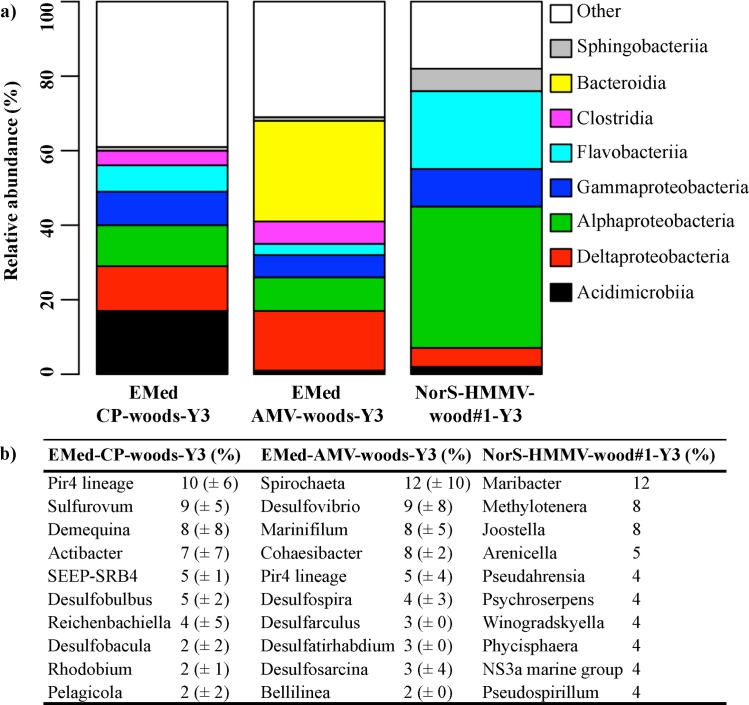
**Taxonomical comparison of bacterial communities at the class (a) and genus (b) level.** Analysis reveal spatial variations in the wood-associated bacterial communities immersed for 3 years between experiments at the Central Province (CP), Amon Mud Volcano (Amon) and Håkon Mosby Mud Volcano (HMMV). (a) Depicts the five and (b) the ten most sequence-abundant classes and genera, respectively, given as percentages. For this analysis sample of wood experiments deployed in the same province were pooled together. See also S6 Table for more details.

Increasing differences with geographic distance between the wood communities was also evident at class level (Fig 6A). Sequences affiliated to Alphaproteobacteria, Flavobacteriia and to certain extent to Gammaproteobacteria had highest relative abundance at NorS-HMMV-wood#1-Y3 after 3y of immersion (Fig 6A and S5 Table). In contrast, Acidimicrobiia and Bacteroidia sequences were most dominant on the wood logs deployed for the same period of time at the Central Pockmark and Amon Mud Volcano, respectively. Sequences belonging to the Deltaproteobacteria and Alphaproteobacteria had similar relative abundance in the experiments at both regions in the Eastern Mediterranean. The *Pir4* lineage (Planctomycetacia) and various genera of the Deltaproteobacteria e.g. SEEP-SRB4, *Desulfobulbus*, *Desulfovibrio*, were among the most abundant genera of the 3y-immersed wooden logs in both regions in the Eastern Mediterranean (Fig 6B and S6 Table). In contrast *Maribacter* and *Joostella* of Bacteroidetes, and *Methylotenera (*Betaproteobacteria) were the most sequence abundant genera in NorS-HMMV-wood#1-Y3 (Fig 6B). Overall, only 3 genera (*Pacificibacter*, *Pseudahrensia*, *Leisingera* of Alphaproteobacteria) were common to all wood experiments over the entire immersion period.

#### Wood-chip and sediment bacterial communities

**Fig 7:**
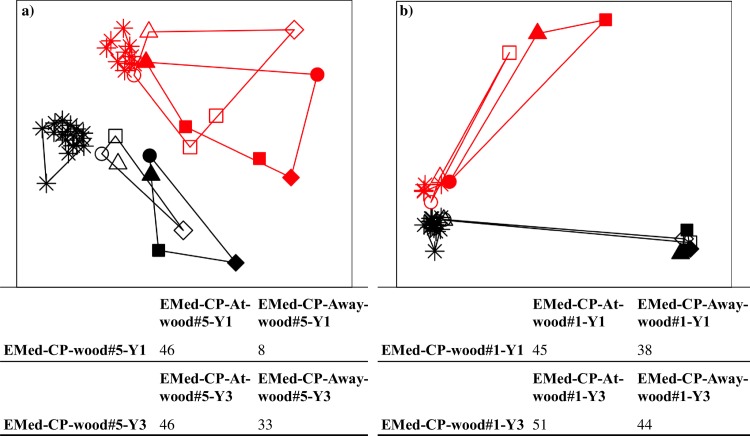
NMDS analysis depicting differences in the structure between wood and sediment bacterial communities. Analysis were performed using Bray-Curtis dissimilarity based on the ARISA dataset. “At wood” (open symbols) and “Away wood” (closed symbols) samples changed with time in (a) EMed-CP-wood#1 and (b) EMed-CP-wood#5 experiments. Communities sampled after 1y of immersion are colored in black and communities after 3y in red. Different symbols of the sediment communities refer to the depth horizon sampled (circle = 0–1 cm, triangle = 1–2 cm, square = 4–5 cm and rhomb = 9–10cm), while wood samples are depicted with an asterisk. Note that topmost surface 0–2 cm “At wood” samples (open symbols) contained variable amounts of wood-chips mixed with sediment. NMDS plot (a) stress = 18% and NMDS plot (b) stress = 7.5%. Percentage of the shared ARISA OTUs between the wood and the sediment “At wood” and “Away wood” samples for the different immersion times is given in the tables below the NMDS panels. See S8 Table for associated results of the ANOSIM test.

NMDS and ANOSIM analyses revealed that wood-associated bacterial communities remained significantly different across all experiments and with time, compared to those in the wood-chip piles, underlying sediments and reference sediments (Fig 7, S8 Table). Over the course of three years only 30–45% of the bacterial genera became common to both wood-associated and wood-chip samples.

In contrast to the wood-associated communities, the bacterial community structure of the wood-chips (0–2 cm layer) and sediments did not significantly change with time (ARISA based data; Fig 7 and S8 Table); however shifts in the relative abundance of the most dominant bacterial classes were observed for both EMed-CP-wood#1_0-2cm and EMed-CP-wood#5_0-2cm experiments (S6 Table). Within the wood-chip layer, the relative sequence number of Alphaproteobacteria and Epsilonproteobacteria decreased, Gammaproteobacteria remained stable, and Deltaproteobacteria and Anaerolineae increased over the course of immersion. The deeper sediment layer (EMed-CP-At_wood#1_5-7cm-Y3), visually not affected by wood-chips, showed similar distribution of the most abundant bacterial classes. Numerous Deltaproteobacteria affiliated to *Desulfarculus*, *Desulfobacterium*, *Desulfobacula*, *Desulfobulbus* and SEEP-SRB4, as well as *Pir4* lineage and *Sulfurovum* were among the ten most abundant genera in the 3y wood-chip samples.

### Influence of wood falls on sediment biogeochemistry

Under the layers of wood chips, high sulphide porewater values (max. 1.2 mM) were detected only in the deeper sediment layers (> 8 cm) 2 years after submergence at EMed-CP-wood#6-Y2 (data not shown). After 3y of immersion (Table 3), and in contrast to measurements performed after 1y of deployment, no free sulphide was detected anymore at any of the other wood-influenced sediments (EMed-CP-At-wood#1-Y3, wood#3, wood#4, wood#5) in the Eastern Mediterranean in 2009. However, a blackened reduced layer, most probably originating from sulphide precipitated with iron, was observed at the boundary between the wood-chips and the sediment surrounding all wood experiments in the Eastern Mediterranean. Also, the sediment sulphate reduction rates were around 1 mmol m^-2^ d^-1^ (Table 3), similar to the rates detected two years before. No anaerobic oxidation of methane was detected in the sediments at any of the investigated sites neither after one or three year of the immersion of the wood experiments, thus SR was most likely related to wood degradation.

**Table 3:** Summary of biogeochemical measurements performed at wood-influenced (0.5 m–“At wood”) and reference sediments away from the wood experiments (> 10 m distance–“Away wood”) in the Eastern Mediterranean after one year of submergence (2007; [14]) and 3 years (2009, this study). For comparison, data from surrounding seafloor sediments were included. The table combines information on the *in situ* Total Oxygen Uptake (TOU), *in situ* and *ex situ* Dissolved Oxygen Uptake (DOU), Oxygen Penetration Depth (OPD), total sulphide flux as determined *ex situ* with microsensors and *ex situ* average Sulphate Reduction (SR) rates integrated over 0–10 cm. n.d. = not determined.

	TOU	DOU	OPD	H_2_S	SR
	mmol m^-2^ d^-1^	mmol m^-2^ d^-1^	mm	mmol m^-2^ d^-1^	mmol m^-2^ d^-1^
**EMed-CP-At-wood#1-Y1**	25*	4.3 ± 0.9	6.7	31.6 ± 6.7	1.3
**EMed-CP-At-wood#1-Y3**	n.d.	3.7 (4.5)	0	n.d.	1.3
**EMed-CP-Away-wood#1 -Y1**	1*	2.3 ± 0.4	6.7	15.5 ± 6.1	2.5
**EMed-CP-Away-wood#1-Y3**	n.d.	0	15.9*	n.d.	0.2
**EMed-AMV-At-wood#3-Y3**	n.d.	4.8	7.1	n.d.	2.4
**EMed-AMV-At-wood#4-Y3**	60*	3.6* (5.8)	6.3* (4.2)	n.d.	1.7
**EMed-AMV-Away-wood#4-Y3**	20*	1.5* (2.6)	>63* (>25)	n.d.	0.1
**EMed-CP-At-wood#5-Y1**	n.d.	4.4 ± 0.5	5	19.3 ± 7.6	2
**EMed-CP-At-wood#5-Y3**	n.d.	6.2	0	n.d.	0.1
**EMed-CP-Away-wood#5-Y1**	n.d.	1.0 ± 0.4	> 32	0	0.1
**EMed-CP-At-wood#6-Y2**	64*	3.8	5.4	n.d.	0.8
**EMed-CP-Away-wood#6-Y2**	16*	n.d.	n.d.	n.d.	n.d.
**EMed-CP-At-wood#7-Y0**	n.d.	0.3	>55	n.d.	0
**EMed-pelagic-sediment**^**1**^	1	0.5	>10	n.d.	n.d.

* = *in situ* measurement. Where both *in situ* and *ex situ* data are available, the latter is placed in parentheses.

^1^ Grünke et al., 2011.

**Fig 8:**
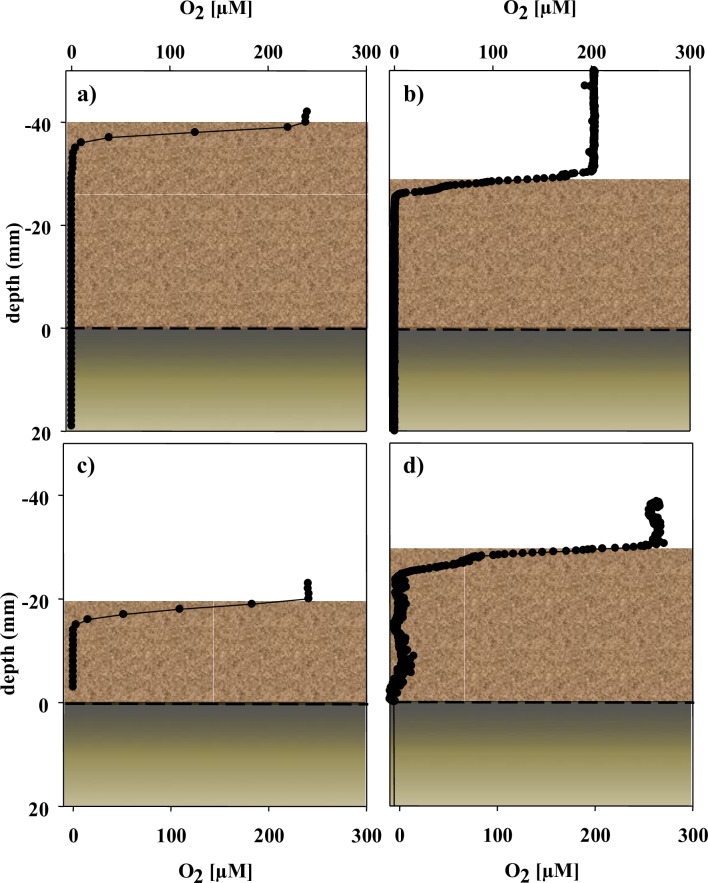
Microsensor measurements of oxygen concentrations at wood influenced sediments. Measurements were performed at EMed-CP-At-wood#1 (a, b) and EMed-CP-At-wood#5 (c, d) sediments after 1 year (a, c) and 3 years (b, d). Oxygen measurement in (b) was performed *in situ* and in (a, c, d) *ex situ*. The sediment surface is indicated with dashed black line, denoting the boarder between the wood chip (brown color) and sediment layer (olive green color).

Similar oxygen consumption rates prevailed at the wood-influenced sediments after 1, 2 and 3 years of deployment at both EMed-CP-wood#1 and EMed-CP-wood#5 experiments, with diffusive oxygen uptake (DOU) of 3.7–6.2 mmol m^-2^ d^-1^ and oxygen penetration depth (OPD) as shallow as 4.6 mm (Table 3 and Fig 8), compared to a penetration depth of > 15.9 mm at the reference site (e.g. EMed-CP-Away-wood#1-Y3). Oxygen was completely consumed within the wood-chip layer at all investigated experiments, i.e. did not reach the underlying sediment (Fig 8). Comparable diffusive oxygen fluxes (3.4–3.6 mmol m^-2^ d^-1^) were detected also in the wood-influence sediments surrounding the sunken wood experiments at the Amon Mud Volcano (EMed-AMV-At-wood#3-Y3, EMed-AMV-At-wood#4-Y3; Table 3).

Total oxygen flux assessed by benthic chamber measurements, including microbial and faunal respiration, measured after 2y (EMed’09-CP-wood#6-Y2) and 3y (EMed’09-AMV-wood#4-Y3) of submergence was more than two times higher (60–63 mmol m^-2^ d^-1^) as compared to the first year of immergence of EMed’09-CP-wood#1-Y1 (Table 3).

Even after 3 years, the influence of the wood at all investigated experiments in the Eastern Mediterranean Sea was confined only to the sediments located in the vicinity of the experiments (approx. ≤ 0.5 m). The impact of the sunken wood logs on these surrounding sediments was evident from the elevated oxygen fluxes (diffusive and total) and shallower penetration of oxygen detected next to the experiments, as compared to the reference sediments located approx. 10 m from the wood experiments (Table 3). At the reference sediments EMed-CP-Away-wood#1-Y3 and EMed-CP-Away-wood#4-Y3 oxygen penetrated deeper than 7 and 63 mm, respectively, and the total benthic oxygen uptake was 3–4 times lower compared to the wood-influenced sediments (Table 3). Correspondingly, diffusive uptake of oxygen of pelagic sediments of the Eastern Mediterranean not impacted by wood falls or methane seepage was almost an order of magnitude lower than in the wood influenced sediments (1 mmol m^-2^ d^-1^).

The DOM molecular composition differed between porewaters of the wood-influenced sites and their respective references. The diversity of DOM compounds (defined as the number of molecular formulae) was higher at EMed-CP-At-wood#5-Y1 compared to its reference site EMed-CP-Away-wood#5-Y1 (S3 Table). Sediments surrounding EMed-CP-wood#1-Y1 displayed a much larger variability in the number of molecular formulae, and results may be confounded by its location at an active seep. Thus, in order to attribute differences in DOM composition to the presence of wood, we focused on sediments around EMed-CP-wood#5-Y1, which was deployed on deep-sea muds not influenced by seepage. Specifically, we compared the DOM composition of surface sediments at the reference site (EMed-CP-Away-wood#5-Y1) with the wood-chip sediment boundary layer (EMed-CP-At-wood#5-Y1), where a 2–3 cm thick layer of wood chips had accumulated on top of the seafloor. Overall, higher relative contributions of unsaturated aliphatic-O rich molecules, peptides and sugars-CHOX formulae were displayed at the EMed-CP-At-wood#5-Y1 wood-influenced site, as well as higher contributions of polyphenols and condensed aromatics (S2A Fig). In the wood-chip sediment boundary layer, 1354–2319 molecular formulae were unique when compared to reference surface sediments, and comprised mainly CHON formulae, as well as sulfur- and phosphorous-containing formulae (S2B and S3 Figs). These were of higher molecular weight and had higher O/C ratios compared to the reference site (S3 Table). Most of these formulae were also absent from deeper sediment layers at the wood-influenced site.

No biogeochemical measurements could be made on sediments associated with Norwegian Sea wood experiments due to limited dive time, however the occurrence of potentially thiotrophic bacterial mats on the sediment surface in contact with the wood logs also indicate the development of sulphidic conditions there, after 2 years of immersion of wood logs. Such bacterial mats occur at the central mud flow area of the HMMV where sulphide fluxes are high, with rates in the range of 43 mmol m^-2^ d^-1^ [35,41].

## Discussion

### Successional stages of wood-associated communities

This study provides evidence that sunken wood logs are highly dynamic ecosystems with substantial succession in the wood-associated communities within the first 3 years of immersion. For wood falls in the Eastern Mediterranean, a succession of wood-boring bivalve species and an apparent increase in the abundances of sipunculids in the wood logs were observed visually. Our qualitative observations of large fauna on the wood logs, but also the biogeochemical and microbiological analyses, support a classification of successional stages for wood falls, similar to what has been previously documented for other type of large organic falls [3,12,15,16,70,71]. Based on the findings of this and other studies [11,20,70–72], four overlapping successional stages in the degradation of wood falls can be proposed for faunal and bacterial communities: 1) a *specialist stage* [73], occurring within the first couple of months of the wood arrival at the sea floor and characterized by invasion of woodborers that initialize the degradation of wood; 2) an *opportunist stage* [6,73], initiated already before the sulphophilic stage and lasting for 1–2 years, with a peak during the main growth of woodborer populations, when detritus-feeders and predatory organisms, e.g. sipunculids, pycnogonids, sea urchins, as well as bacterivores get attracted by the accumulation of biomass; 3) a *sulphophilic stage* (duration > 1–2 years)[14,27,28,74], during which enhanced cellulose degradation leads to sulphidic conditions and a colonization by chemosynthetic organisms i.e. *Idas* sp., siboglinids, takes place; 4) a *senescence stage*, initiated after the third year of degradation, characterized by the disintegration of wood logs, the dispersal and burial of the wood-chips, as well as a decline of numbers of large faunal organisms including reduction of woodborer biomass. However, the type of colonizers and the temporal development of the stages may strongly depend on several factors, such as the type of wood, the size of the wood [73] and the biogeographical area of wood deposition.

Symbiotic wood-boring bivalves such as the deep-sea genus *Xylophaga* sp. [75] play a key role in the degradation of wood in deep-sea marine environments, and hence, are very important for the ecology and biogeochemistry of wood fall ecosystems [11,14,76]. Our experiments comply with the results of a previous study of minimum shell sizes of pioneer communities [77], and suggest that larvae of the wood-boring bivalves are able to disperse and settle on the sunken wood at sizes >0.3 mm (S1 Fig), and that the adult bivalve stage develops and reproduces within less than a year, potentially within a few months. Interestingly, we were able to observe shifts in the occurrence of wood-boring species within a period of three year of submergence. In the Eastern Mediterranean Central Province, *Xylophaga dorsalis* dominated the wood-experiments, as well as other small colonization devices [29], but declined in abundance already after the first year of deposition, and was almost substituted by a different and less abundant Xylophagainae species *Xylophaga brava* (Table 2). For long, only one wood boring species (*Xylophaga dorsalis*) was known from the Mediterranean Sea. However, just recently a study on wood falls in a submarine canyons in Blanes discovered three unidentified species of *Xylophaga* spp. that co-colonized sunken wood logs and had variable abundances with time [67,78]. These findings suggest that different species of wood boring bivalves have adopted different ecological strategies, e.g. certain species initializing the degradation of wood, forming openings through the bark, borrowing inside the wood, producing wood chips and fecal matter as well as biomass, and others being important in the later stages of the degradation. Moreover, potentially more than one species of wood-boring bivalve might be relevant for the complete degradation of wood falls in the deep Eastern Mediterranean sea. Another possibility that can explain the patterns observed in this study could be the observation that different *Xylophaga* species have variable modes of spawning i.e. periodic versus continuous [79], which could have an impact on the structure of the pioneering colonizer communities. To better understand the commonality and causality of such events continuous long-term monitoring should be employed that would investigate the ecology of the host species as well as the metabolic potentials of their symbionts.

Cellulose is the major component of plant and woody material that reaches the deep sea floor, and, after chitin, represents the second most important carbohydrate in marine environments [14]. Degradation of cellulose and its degradation products is a complex process that often proceeds through aerobic and anaerobic phases [22], and is hence mediated by complex fungal and bacterial communities including (an)aerobic cellulolytic bacteria, fermenters, sulphate-reducers and sulphide–oxidizers [14,25]. Fungi are the main decomposers of wood on land, but little is known as to their role in marine environments. Some studies assume that they are less relevant compared to bacteria in the wood degradation process [24,25]. However, different fungi species (mainly belonging to Ascomycetes, Basidiomycetes, and Deuteromycetes) have been found associated to or growing on sunken wood as well as other cellulosic material in the ocean [80,81], but their role in deep-sea sunken wood ecosystems is still unclear and should be addressed in future studies. Repeated investigation of the same submerged wood experiments over the course of three years in this study revealed a statistically significant temporal shift in the structure of wood-associated bacterial communities (Fig 3). This expands the findings of previous studies, which have identified a tentative grouping according to time of immersion [24,82], as well as a shift of terrestrial to marine signature in the bacterial community structure over the course of one year [14]. Despite the same duration of immersion (3 years), the Central Pockmark wood logs were substantially more degraded at any sampling period compared to Norwegian Sea logs (Fig 1). Possible factors responsible for the observed contrasting patterns could be the difference in the main wood-boring bivalves found at the different experiments and/or differences in the temperature of the environment where the experiments were deployed, i.e.– 1°C in the Norwegian sea vs. 14°C in the Eastern Mediterranean. The cold temperatures could reduce the metabolic rates of wood-degrading organisms and thus the overall wood degradation rate.

Here, a strong temporal shift in the bacterial community structure was observed in all wood logs between two and three years of deposition, but not in the surrounding sediments including the wood-chip layer (Figs 3 and 7). This suggests that sunken wood logs are temporally dynamic ecosystems, most likely due to the rapid faunal succession described above. Sequences affiliated to known Deltaproteobacteria sulfur-reducers (*Desulfobulbus*, SEEP-SRB4) as well as sulphide-oxidizers of the Epsilonproteobacteria (*Sulfurovum*) increased in abundance over time at the wood experiments in the Central Province. Concomitantly, the abundance of Bacteroidetes (*Tenacibaculum*, *Maribacter* and *Leadbetterella*) potentially involved in aerobic degradation of cellulose or its derivatives [83–85] declined (Fig 4). Similarly, a previous study has shown a dominance of aerobic cellulose-degraders vs. sulphate-reducers in short-term and long-term wood experiments, respectively [25]. This is likely a response to changes in faunal activity (see also [26]) and succession, and to the depletion of oxygen after which cellulose is predominantly degraded anaerobically with sulphate, producing sulphide [22]. Such a functional succession was also observed at the wood experiments deployed in the Norwegian Sea, with increase of sulphate-reducers of *Desulfobacterales* and *Desulfomonadales* with immersion time and a shift in the putative aerobic cellulose-degraders i.e. dominance of *Alteromonadales* (Gammaproteobacteria) during earlier phases and by *Myxococcales* (Deltaproteobacteria) and *Saprospiraceae* (Sphingobaceria) and during later stages of immersion. Degradation of wood proceeds non-homogenously and likely different parts remain oxic while others become anoxic, which in turn promotes the formation of multiple niches, as suggested also earlier [25].

### Influence of sunken wood on seafloor biogeochemical processes

Whale falls have a long-term (several decades) impact on the geochemistry of sediments surrounding the falls, because of the massive input of organic carbon in the form of lipids, causing the production of sulphide and methane [4,10,13,70]. In contrast to lipid and protein-based organic matter, wood cellulose is much more difficult to degrade due to the high degree of polymerization, rigid microfibril organization, insolubility in water and association to lignin. Hence, very few highly specialized microorganisms can metabolize cellulose via production of cellulases. The wood-chip sediment boundary layer has also previously been shown to be an area of strongly elevated DOC concentrations [14], indicating cellulose degradation under anoxic conditions. In the present study we were able to additionally identify a molecular DOM signature unique to porewaters at the wood-influenced sediment site (EMed-CP-At-wood#5-Y1), when compared to its reference (S2 Fig). Molecular groups generally associated with fresh organic matter input were more abundant at the wood-influenced site. At the same time higher contributions of polyphenols and condensed aromatics, usually products of degradation [86], were observed, confirming an active degradation of wood-derived organic matter at these depths. This was also indicated by the higher molecular weights observed in the wood-chip layer compared to the reference site (as expected for wood-derived organic matter), which declined in the wood-chip sediment transition zone, suggesting a decomposition of the wood-derived material (S3 Table). In addition, most of the CHON compounds unique to the wood-influenced sediments were lignin- or cellulose-like in their H/C and O/C ratios (H/C = 0.7–1.5, O/C = 0.1–0.67; [87,88] (S3 Fig) and were characterized by higher unsaturation and aromaticity, indicative of terrestrial plant-derived material [88]. These groups of compounds may thus serve as indicators for the deposition and degradation of wood at the seafloor. This signature in the porewater is likely restricted to a radius of 0.5–1 m around the wood, corresponding to the area covered by wood chips. The temporal evolution and persistence of this signature remain to be assessed in future studies.

Only recently, we could show by *in situ* quantification that sunken wood logs promoted sulphide production within a year, in the wood-chip layer accumulated on top of the sediments [14]. This study indicates wood-fueled sulphide production for > 3 years, also evident by a bacterial community dominated by the sulphide-oxidizer *Sulfurovum* as well as numerous and diverse sulphate-reducers of the Deltaproteobacteria (S5 and S6 Tables). These results explain how some types of thiotrophic chemosynthetic life can find a niche and a stepping-stone on wood falls [18]. However, in contrast to whale falls [4], the influence of the sunken wood logs on the sediment geochemistry did not expand laterally beyond a few meters distance from the fall, even after 3y of immersion. The feeding behavior and succession of wood-boring bivalves may be important for the areal impact of sunken wood, as well as for the associated biogeochemical processes. At whale falls, sharks and hagfish take bites from the carcass and distribute blubber in the vicinity [3], but such larger, mobile predators are missing from wood falls. Also the type of wood-boring bivalve may change the area under influence: here we observed how the calcifying wood-boring bivalve responsible for the degradation of Norwegian Sea sunken wood logs did not produce massive amounts of wood chips, but formed carbonaceous tubes gluing the wood together, despite of its progressing degradation.

It still remains unknown if cellulose degradation in sediments can lead to methane production as observed at whale falls [10]. A prerequisite would be the complete consumption of sulphate in the porewaters and the suppression of substrate competition between sulphate-reducing bacteria and methanogens [89]. In the experiments conducted here, we could not observe a complete consumption of sulphate in the sediment porewaters around the wood falls, hence, excluding the development of methanogenic zones.

### Spatial variations of wood-associated communities

Similar to cold seeps and hydrothermal vents, all types of large organic falls have a highly fragmented distribution and represent island-type habitats in the vast oligotrophic deep sea [3]. Given the fragmented and isolated nature of these habitats, it is important to better understand the dispersal patterns, and hence the biogeography and interconnectivity of biological communities associated with these habitats [19,90–92]. Previous studies focusing primarily on the biogeography of vent, and to a certain extent on seep fauna, have identified six biogeographic provinces, determined by the geographic location of the ocean basins and their isolation along the mid-ocean ridge system [93,94]. The major factors responsible for structuring seep fauna were depth, and to a lesser extent geographic distance [95]. However, research on the biogeography of organic fall communities is still in its infancy, due to limited observations [3,19]. Similar to cold seeps, water depth has been suggested to influence the distribution of whale fall communities [70,71,96,97]. In this study, wood experiments located at two different cold seeps in the Eastern Mediterranean Sea (CP and AMV; 130 km distance) had more or less identical abundant taxa i.e. wood-boring bivalves *Xylophaga brava*, chemosynthetic mussels *Idas modiolaeformis* (seep-type fauna) and sipunculids, as well as several types of background megafauna (*Asterechinus elegans* sea urchins and *Bathynectes piperitus* crabs) overlapping with those inhabiting the near-by seep habitats. The wood experiments deployed in the Norwegian Sea at the Håkon Mosby Mud Volcano, also attracted fauna from the near-by seeps at HMMV, namely the chemosynthetic siboglinid tubeworm *Sclerolinum contortum*, a few polychaetes, as well as pycnogonids. Hence, the fauna was distinct from the Eastern Mediterranean experiments, including a different type of woodborer species *Xyloredo*. The biogeography of wood-boring bivalves is not fully resolved today [67]. *Xyloredo ingolfia* was previously identified in a colonization experiment filled with wood substrate at the HMMV [29]. The Eastern Mediterranean woodborer *Xylophaga dorsalis* has been reported also from cold northern waters i.e. offshore of Iceland [72] and Norway [98], however no specimens of this species were found to colonize the Norwegian Sea wood experiments in this study. Some thyasirid bivalves have been previously found at HMMV and at other seeps of the Norwegian Sea [42], but neither these nor other chemosynthetic bivalves were recovered from the wood experiments in the Norwegian Sea. We found chemosynthetic siboglinid tubeworms attached to the bark of the HMMV experiments which are indicative of sulphide production from wood, because their symbionts are thiotrophs [99]. Several other studies had reported siboglinids associated with sunken wood, indicating that wood falls might represent a natural habitat for these chemosynthetic organisms [29,42,98]. Although up to twelve species of sipunculids have been found to live in the Arctic Ocean (http://www.arcodiv.org/seabottom/worms/Sipunculids.html), none were previously reported from wood experiments in northern waters. The results of this study suggest that the wood-associated fauna investigated in this study belong to two different biogeographic provinces, i.e. the Eastern Mediterranean and the Norwegian Sea. We found evidence for faunal exchange between seeps and wood falls within each province, but only limited dispersal across ocean basins, due to biogeographical barriers [93].

This study showed that also bacterial communities of sunken wood logs located at the same cold seep site were overlapping and had highly similar structure with similar abundances of Deltaproteobacteria (12–16%) and Alphaproteobacteria (9–11%; Fig 6A and S5 Table). Also, we found evidence of bacterial dispersal between distant seep sites within one region, i.e. between AMV and CP (Fig 6; S5 Table). However, although sequences affiliated to sulfur-reducers were among the most abundant types at the Central Province and Amon Mud Volcano, wood logs of these two regions selected for different taxonomical types capable of performing sulphate reduction i.e. SEEP-SRB4, *Desulfobulbus* and *Desulfobacula* at the Central Province, versus *Desulfovibrio*, *Desulfospira*, *Desulfarculus*, and *Desulfatirhabdium* at the AMV (Fig 6). The comparison of bacterial community composition between the two biogeographical regions showed that like the fauna, the bacterial community of the wood logs deployed at the HMMV in the Norwegian Sea was distinct from the logs deployed in the Eastern Mediterranean Sea.

Accordingly, a trend of significant increase of dissimilarities in the bacterial communities with increasing geographic distance was identified (Fig 6). Similar patterns were also shown for other fragmented ecosystems, i.e. cold seeps [49], but for sunken wood logs no evidence had been found so far that the geographic location played a role in structuring wood-associated communities [24]. The present results support the hypothesis of dispersal limitations across provinces also for wood-associated bacteria [100,101], but cannot resolve whether this is due to different temperature regimes or due to distance and geographical barriers.

### Sunken wood host distinct bacterial communities at the deep- sea floor

Results of this study reveal that wood falls are highly dynamic and variable ecosystems, both spatially and temporarily, and that they are colonized by a large diversity of bacterial groups providing similar biogeochemical functions in different regions (see also [73]). Thus, few genera–only 6% and 24% of all detected in this study—were found in common to the wood experiments deployed in the Eastern Mediterranean and the Norwegian Sea, respectively, during the entire immersion period of three years. These included among others the *Pir4* lineage (Planctomycetes), *Demequina*, *Sulfurovum*, *Tenacibaculum*. The *Pir4* lineage has also been found previously at other wood falls [82], and represented the most abundant genus with 7% of all wood-related sequences in our whole dataset. Based on these findings *Pir4* lineage likely plays a crucial role at wood fall ecosystems, however its specific biogeochemical functions remain unknown. Within the Alphaproteobacteria, we detected a core wood community–i.e. bacterial groups present in all wood experiments during all sampling periods, comprised of microorganisms belonging to the genera *Pacificibacter*, *Pseudahrensia*, *Leisingera*, of which the latter two are affiliated to families with known cellulose-degraders [102,103]. Likely these genera are among the first ones to colonize wood in marine environments, as they were already detected in wood logs immerged for 1-day and 1-month, but with lower relative sequence abundance.

Additionally, some wood-associated bacterial taxa identified here had a wide-range distribution, as they were also previously found in association to natural or artificial sunken wood logs in the Western Mediterranean and the Pacific Ocean. These include putative cellulose-degraders *Maribacter*, *Spirochaeta*, *Methylotenera*, the sugar-fermenting *Marinifilum*, the aerobic heterotroph *Pseudospirillum*, *Pir4* lineage, as well as numerous sulphate-reducing genera of the Desulfobacteraceae [25,27] and Desulfovibrionaceae [25,27,82,104]. In addition, here we report on bacteria not previously detected in other wood fall experiments, which were highly abundant in our deployments, and which might have an important role in the degradation of wood: *Tenacibaculum* and *Leadbetterella* of Bacteroidetes, *Novosphingobium* (Alphaproteobacteria), *Demequina* (Actinobacteria, see also [14]) and *Christensenella* of Firmicutes. These genera were previously shown to be able to utilize cellulose derivatives, or to be closely related to cellulolytic taxa, isolates or sequences from wood falls and shipwrecks [84,85,102,103,105,106].

Across all experiments, and even after 3 years of immersion, a significant difference in the taxonomical composition of bacterial communities was detected between the sunken wood-associated communities and the surrounding seafloor communities. Typical deep-sea floor communities in the Eastern Mediterranean were comprised of Beta- and Gammaproteobacteria [49,101] while sunken wood logs deployed in the same area were dominated by Acidimicrobiia, Bacteroidetes, Delta- and Alphaproteobacteria. Similarly, the sunken wood community in the Norwegian Sea was mostly dominated by Alphaproteobacteria and Flavobacteriia, in contrast to the dominance by Gamma- and Deltaproteobacteria in deep-sea sediments from that area [107]. Hence, sunken wood represents not only a temporary energy source to deep-sea communities, but also enhance their diversity in general.

## Supporting Information

S1 Text Material and Methods: Dissolved Organic Matter analyzes. (PDF)

S2 Text Wood tiles in nets experiment. (PDF)

S1 Table Location of wood experiments in the Norwegian Sea and the Eastern Mediterranean Sea, description of the benthic habitats, date of deployment and samplings events. Deoxyribonucleic acid (DNA), Anaerobic Oxidation of Methane (AOM), Sulphate Reduction (SR), benthic chamber (CHAM) and microprofiler (MICP).
(PDF)

S2 Table 454 MPTS-derived sample characteristics of v6-extracted datasets. (PDF)

S3 Table Overview of the composition of dissolved organic matter at wood colonization experiments EMed-CP-wood#5 and EMed-CP-wood#1 after 1 year of immersion. (PDF)

S4 Table Temporal variations of wood-associated bacterial communities. Analysis of Similarity (ANOSIM), testing for significant difference among wood experiments in the Eastern Mediterranean Sea immersed for variable periods of time. ***p < 0.001, **p < 0.01, p < 0.05 after Bonferroni correction. Analyses were based on the ARISA dataset.
(PDF)

S5 Table Temporal comparison of the ten most sequence-abundant bacterial classes of wood samples immersed for different periods of time. Numbers represent percentages of the relative sequence abundance for each wood experiment. Analyses are based on the v6-extracted 454 MPTS dataset.
(PDF)

S6 Table Temporal comparison of the twenty most sequence abundant genera of wood-associated (a, b), wood-chip (c) and sediment (c) samples immersed for 1 to 3 years. Relative sequence abundance, given as percentages, is shown in parenthesis.
(PDF)

S7 Table Analysis of similarity test (ANOSIM), based on Bray-Curtis dissimilarities. Testing for significant differences in the bacterial community structures between wood experiments deployed for 3 years at spatially distant and close seeps. Asterisk denotes the level of statistical significance (p = 0.001). Analyses were based on the ARISA dataset. Similarity between wood bacterial community structures significantly decreased with increasing geographic distance between the wood experiments (454 data Mantel r = 0.6, p = 0.01, ARISA data Mantel r = 0.5, p = 0.01).
(PDF)

S8 Table Analysis of similarity test (ANOSIM), based on Bray-Curtis dissimilarities. Testing for significant differences in the bacterial community structures between wood-associated and sediment (wood-impacted “At wood” and reference “Away wood”) samples at (a) EMed-CP-wood#1 and (b) EMed-CP-wood#5 experiments immersed for 1y and 3y. ***p < 0.001, **p < 0.01, p < 0.05 after Bonferroni correction; (*) only significant without Bonferroni correction. Analyses were based on the ARISA dataset.
(PDF)

S1 Fig Photos showing wood-boring bivalves colonizing the wood tiles of the nets with 9 mm (a) and 0.3 mm (b) mesh size. (c) wood tiles in the net with the smallest mesh size (0.05 mm) were no populated by wood-boring bivalves. NMDS (d) and ANOSIM analyzes (f) revealing significant differences in the bacterial community structure between wood tiles of the different nets. (f) ANOSIM R values are shown in the lower and Bonferroni corrected p-values in the upper triangle. Percentages of shared OTUs between the wood tiles of the different nets are displayed in (e).
(PDF)

S2 Fig Relative contributions of molecular DOM compounds at EMed-CP-wood#5 (lower bars) after one year of immersion compared to its reference (upper bars). a) DOM molecular formulae observed in porewaters indicating the elemental composition in the different samples, and b) Major molecular groups at EMed-CP-wood#5 and its reference site. Bars on the right indicate samples that were directly compared, i.e. surface sediments at the reference site and the wood-chip sediment boundary layer at the wood experiment, where a 2–3 cm thick layer of wood chips had accumulated on top of the seafloor.
(PDF)

S3 Fig van Krevelen diagram of H/C and O/C ratios displaying molecular formulae that were unique (not present at 0–1 cm reference site) to the wood-chip sediment transition zone at EMed-CP-wood#5. Unique formulae mainly belonged to groups containing CHON (1668 formulae), sulfur (958 formulae) and phosphorous (237 formulae); the latter two also include the combination of S and P with other elements such N.
(PDF)

S4 Fig 3D-NMDS analysis based Bray-Curtis dissimilarity, depicting spatial variations in the wood bacterial community structure between Central Province (black), Amon Mud Volcano (red) and Håkon Mosby Mud Volcano (green) wood experiments immersed for 3 years. Samples are color coded according to location of wood experiments. Stress = 14%.
(PDF)
