# Supplementary material for: Temporal and Spatial Variations of Bacterial and Faunal Communities Associated with Deep-Sea Wood Falls
Source: PLoS One. 2017 Jan 25;12(1):e0169906. doi: 10.1371/journal.pone.0169906 (PMC5266260; doi:10.1371/journal.pone.0169906)
Supplement: S1 Text — (PDF) [file pone.0169906.s001.pdf]

S1 Text

Material and Methods: Dissolved Organic Matter analyzes. Molecular information was evaluated based on the contribution of different elements (CHO, CHON, CHOS, CHOP and CHONS formulae) and by the intensity weighted average (wa) of the Double Bond Equivalents (DBE_{wa}) and Aromaticity Index ($AImod_{wa}$) and elemental ratios H/C_{wa} and O/C_{wa} (1–3). Additionally, molecular formulae were associated with different molecular groups in order to provide an overview on the molecular composition of pore water samples (4,5). Molecular groups considered: 1) highly unsaturated compounds (AImod<0.5 and H/C<1.5); 2) polyphenols (0.67 ≥ AImod>0.5); 3) condensed aromatics or dissolved black carbon (DBC, AImod ≥ 0.67); 4) formulae of unsaturated aliphatics (1.5< H/C <2, O/C <0.9, N=0); 5) peptide molecular formulae (1.5< H/C<2, O/C<0.9 and N>0); 6) saturated fatty acids (H/C>2, O/C<0.9), without (saturated FA) or with heteroatoms (N, S or P) (saturated FA-CHOx); 7) formulae of sugars (O/C>0.9), without (sugars) and with heteroatoms (sugars-CHOx). Furthermore, differences in composition regarding oxygen rich (O-rich) or poor (O-poor) compounds, were also evaluated.

References:

1. McLafferty FW, Turecek F. Interpretation of mass spectra. Fourth Edi. Vetter W, editor. Mill Valley, California; 1993. 379 p.
2. Koch BP, Dittmar T. From mass to structure: an aromaticity index for high-resolution mass data of natural organic matter. Rapid Commun Mass Spectrom. 2016;30(1):250.
3. Koch BP, Dittmar T. From mass to structure: an aromaticity index for high-resolution mass data of natural organic matter. Rapid Commun Mass Spectrom. 2006;20(5):926–32.
4. Seidel M, Beck M, Riedel T, Waska H, Suryaputra IGNA, Schnetger B, et al. Biogeochemistry of dissolved organic matter in an anoxic intertidal creek bank. Geochim Cosmochim Acta. 2014;140:418–34.
5. Rossel PE, Bienhold C, Boetius A, Dittmar T. Dissolved organic matter in pore water of Arctic Ocean sediments: Environmental influence on molecular composition. Org Geochem. 2016;97:41–52.
