# Supplementary material for: Temporal and Spatial Variations of Bacterial and Faunal Communities Associated with Deep-Sea Wood Falls
Source: PLoS One. 2017 Jan 25;12(1):e0169906. doi: 10.1371/journal.pone.0169906 (PMC5266260; doi:10.1371/journal.pone.0169906)
Supplement: S2 Text — (PDF) [file pone.0169906.s002.pdf]

**S2 Text**

**Wood tiles in nets experiment. Material and methods:** Wood tiles (10 x 10 x 1 cm) of Douglas fir were placed in nets with three different mesh sizes: 9 mm, 0.33 mm and 0.05 mm. Each net contained nine identical wood cubes. Nets were attached at the frame of a lander and deployed at the Håkon Mosby Mud Volcano in the Norwegian Sea (1.5 m above the seafloor) in 2009 during the ARKXXIV/2 cruise. Nets were recovered after one year of immersion (in 2010, during the MSM16 cruise), wood tiles were cut open and four subsamples were taken from three wood tiles from each net for bacterial community analyzes by Automated Ribosomal Intergenic Spacer Analysis (ARISA). Molecular and statistical analyzes were performed following the same procedures described in the main text.

**Results:** The wood tiles showed the beginning of degradation of the woods by wood-boring bivalves after 1 year, indicated by presences of borings (S1 Fig). Most borings were observed in the 9 mm net-tiles ($71 \pm 35$, $n = 9$) (S1a Fig), less in the 0.33 mm ($27 \pm 28$, $n = 9$) (S1b Fig) and none in the 0.05 mm net (S1c Fig). In addition, few living specimens of wood-boring bivalves were observed in some of the wood tiles of the 9 mm and 0.33 mm nets.

Nonmetric multidimensional scaling analyzes (NMDS) and Analysis of similarity test (ANOSIM) analyses revealed that wood tiles of the three nets (different mesh-sizes) had significantly different but overlapping bacterial community structures (S1d Fig) (Bonferroni corrected p-value $< 0.02$; S1f Fig). The wood tiles of the net with the biggest mesh size (9 mm) were the most dissimilar compared to the other nets. All nets had between 36 and 40 % OTUs in common (S1e Fig).
