# Supplementary material for: Temporal and Spatial Variations of Bacterial and Faunal Communities Associated with Deep-Sea Wood Falls
Source: PLoS One. 2017 Jan 25;12(1):e0169906. doi: 10.1371/journal.pone.0169906 (PMC5266260; doi:10.1371/journal.pone.0169906)
Supplement: S1 Table — Deoxyribonucleic acid (DNA), Anaerobic Oxidation of Methane (AOM), Sulphate Reduction (SR), benthic chamber (CHAM) and microprofiler (MICP). (PDF) [file pone.0169906.s003.pdf]

Location	Wood experiment	Sampling habitat	Sample/Measurement	Pangaea Label ID
**Eastern Mediterranean; Central Province**	**EMed-CP-wood#1**	wood	DNA	MSM13/3_918-1_WOOD1; -2; -3; -4
		wood-influenced sediment	AOM;SR	MSM13/3_899-1_PUC16; MSM13/3_899-1_PUC21; MSM13/3_899-1_PUC10
			porewater	MSM13/3_899-1_PUC28
			DNA	MSM13/3_899-1_PUC21
			CHAM	MSM13/3_899-1_CHAM1
			MICP	MSM13/3_918-1_MICP1
		reference sediment	AOM;SR	MSM13/3_899-1_PUC13; MSM13/3_899-1_PUC14; MSM13/3_899-1_PUC21
			porewater	MSM13/3_899-1_PUC25
			DNA	MSM13/3_899-1_PUC13
			CHAM	MSM13/3_899-1_CHAM2
			MICP	MSM13/3_918-1_MICP2
	**EMed-CP-wood#2**	wood	DNA	MSM13/3_899-1_WOOD1; -2
**Eastern Mediterranean; Amon Mud Volcano**	**EMed-AMV-wood#3**	wood	DNA	MSM13/3_962-1_WOOD1; -2; -3; -4; -9
		wood-influenced sediment	AOM;SR	MSM13/3_962-1_PUC4; MSM13/3_962-1_PUC27; MSM13/3_962-1_PUC31;
			porewater	MSM13/3_962-1_PUC24
			DNA	MSM13/3_962-1_PUC7
	**EMed-AMVwood#4**	wood	DNA	MSM13/3_944-1_WOOD1; -2; -3
		wood-influenced sediment	AOM;SR	MSM13/3_944-1_PUC2; MSM13/3_944-1_PUC20; MSM13/3_944-1_PUC21
			porewater	MSM13/3_944-1_PUC9
			DNA	MSM13/3_944-1_PUC22
			CHAM	MSM13/3_962-1_CHAM7
			MICP	MSM13/3_947-1_MICP6
		reference sediment	AOM;SR	MSM13/3_962-1_PUCˇ; MSM13/3_962-1_PUC14; MSM13/3_962-1_PUC16;
			porewater	MSM13/3_962-1_PUC20
			DNA	MSM13/3_962-1_PUC22
			CHAM	MSM13/3_962-1_CHAM8
			MICP	MSM13/3_947-1_MICP6

Location	Wood experiment	Sampling habitat	Sample/Measurement	Pangaea Label ID
**Eastern Mediterranean; Central Province**	**EMed-CP-wood#5**	wood	DNA	MSM13/3_976-1_WOOD4
		wood-influenced sediment	AOM;SR	MSM13/3_976-1_PUC11; MSM13/3_976-1_PUC19; MSM13/3_976-1_PUC27
			porewater	MSM13/3_976-1_PUC30
			DNA	MSM13/3_976-1_PUC115
		reference sediment	AOM;SR	MSM13/3_976-1_PUC10; MSM13/3_976-1_PUC22; MSM13/3_976-1_PUC23
			porewater	MSM13/3_976-1_PUC117
			DNA	MSM13/3_976-1_PUC24
	**EMed-CP-wood#6**	wood	DNA	MSM13/3_976-1_WOOD2
		wood-influenced sediment	AOM;SR	MSM13/3_918-1_PUC30; MSM13/3_918-1_PUC16; MSM13/3_918-1_PUC13
			porewater	MSM13/3_918-1_PUC24
			DNA	MSM13/3_918-1_PUC16
			CHAM	MSM13/3_976-1_CHAM14
		reference sediment	CHAM	MSM13/3_976-1_CHAM15
	**EMed-CP-wood#7**	wood	DNA	MSM13/3_976-1_WOOD6
		wood-influenced sediment	DNA	MSM13/3_976-1_PUC24
**Norweigian Sea; Haakon Mosby Mud Volcano**	**HMMV-wood#1**	wood	DNA	PS74/183-1_WOOD-2; -5; -6
		wood-influenced sediment		MSM16/2_844_WOOD1; -2; -3
	**HMMV-wood#2**	wood	DNA	PS74/183-1_WOOD-4; -8; -9;

[3] *RV Maria S Merian; ROV Quest 4000*
[4] *RV Polarstern; ROV Quest 4000*
[5] *RV Maria S Merian; ROV Genesis*
