# Supplementary material for: Temporal and Spatial Variations of Bacterial and Faunal Communities Associated with Deep-Sea Wood Falls
Source: PLoS One. 2017 Jan 25;12(1):e0169906. doi: 10.1371/journal.pone.0169906 (PMC5266260; doi:10.1371/journal.pone.0169906)
Supplement: S2 Table — (PDF) [file pone.0169906.s004.pdf]

	Number of sequences	Number of $OTU_{0.03}$	Number of $OTU_{0.03}$ singletons	Relative number $OTU_{0.03}$ singletons (%)
**EMed-CP-wood#1-Y1**	4602	1240	602	49
**EMed-CP-wood#1-Y3**	3111	281	69	25
**EMed-CP-wood#2-Y1**	3695	913	452	50
**EMed-CP-wood#2-Y3**	8661	1082	499	46
**EMed-CP-wood#5-Y1**	7280	1632	800	49
**EMed-CP-wood#5-Y3**	6603	906	469	52
**EMed-CP-wood#6-Y0**	2086	733	396	54
**EMed-CP-wood#6-Y2**	19358	1612	842	52
**NorS-HMMV-wood#1-Y2**	5533	105	45	43
**NorS-HMMV-wood#1-Y3**	10699	657	334	51
**EMed-CP-At-wood#1-0-2cm-Y1**	5055	1048	398	38
**EMed-CP-Away-wood#1-0-2cm-Y1**	4883	1612	758	47
**EMed-CP-At-wood#1-0-2cm-Y3**	9250	926	383	41
**EMed'07-CP-At-wood#5-0-2cm-Y3**	3957	1746	990	57
**EMed-CP-Away-wood#5-0-2cm-Y1**	5232	1899	873	46
**EMed-CP-At-wood#1-5-7cm-Y3**	6284	357	114	32
**EMed-CP-At-wood#5-0-2cm-Y3**	10093	708	210	30
