# Supplementary material for: Temporal and Spatial Variations of Bacterial and Faunal Communities Associated with Deep-Sea Wood Falls
Source: PLoS One. 2017 Jan 25;12(1):e0169906. doi: 10.1371/journal.pone.0169906 (PMC5266260; doi:10.1371/journal.pone.0169906)
Supplement: S3 Table — (PDF) [file pone.0169906.s005.pdf]

**Wood experiment**	**Sediment depth (cm)**	**Number of molecular formulae**	**Molecular weight (MW_{wa})**	**H/C_{wa} ratio**	**O/C_{wa} ratio**	**Aromaticity index ($AImod_{wa}$)**	**Relative abundance of carboxyl-rich alicyclic molecules (%)**
EMed-CP-Away-wood#5-Y1	0-1	834	391.5	1.35	0.40	0.23	63.4
	1-2	n.a.	n.a.	n.a.	n.a.	n.a.	n.a.
	2-3	598	388.6	1.33	0.40	0.24	68.1
EMed-CP-At-wood#5-Y1	0-1	879	398.9	1.28	0.46	0.25	67.1
	1-2	1811	410.6	1.25	0.48	0.26	64.4
	2-3*	2096	405.6	1.29	0.48	0.24	59.1
	3-4*	3115	394.3	1.34	0.47	0.23	54.2
	4-5	n.a.	n.a.	n.a.	n.a.	n.a.	n.a.
	5-11	2065	405.1	1.33	0.42	0.24	60.6
EMed-CP-Away-wood#-Y1	0-1	4188	420.0	1.29	0.47	0.24	52.8
	1-2	1578	409.1	1.29	0.47	0.24	61.8
	2-3	1305	390.6	1.31	0.47	0.23	60.9
EMed-CP-At-wood#1-Y1	0-1	3666	418.1	1.27	0.47	0.25	54.5
	1-2	3792	422.0	1.26	0.47	0.25	55.1
	2-3	4520	409.3	1.30	0.47	0.24	51.5
	3-4*	1270	410.4	1.26	0.47	0.25	66.9
	4-5*	2418	417.1	1.24	0.48	0.26	60.3
	5-6	884	410.6	1.26	0.48	0.25	69.2
	6-7	1651	410.1	1.27	0.47	0.25	62.7

** The depth layers marked with an asterisk indicate the transition zone between wood-chips and sediment*
