# Supplementary material for: Temporal and Spatial Variations of Bacterial and Faunal Communities Associated with Deep-Sea Wood Falls
Source: PLoS One. 2017 Jan 25;12(1):e0169906. doi: 10.1371/journal.pone.0169906 (PMC5266260; doi:10.1371/journal.pone.0169906)
Supplement: S4 Table — Analysis of Similarity (ANOSIM), testing for significant difference among wood experiments in the Eastern Mediterranean Sea immersed for variable periods of time. ***p < 0.001, **p < 0.01, p < 0.05 after Bonferroni correction. Analyses were based on the ARISA dataset. (PDF) [file pone.0169906.s006.pdf]

	EMed-CP-wood#1-Y1	EMed-CP-wood#2-Y1	EMed-CP-wood#5-Y1	EMed-CP-wood#6-Y1	EMed-CP-wood#1-Y3	EMed-CP-wood#2-Y3	EMed-CP-wood#5-Y3
**EMed-CP-wood#2-Y1**	0.5**						
**EMed-CP-wood#5-Y1**	0.3**	0.45**					
**EMed-CP-wood#6-Y1**	1**	1**	1**				
**EMed-CP-wood#1-Y3**	1**	1**	1**	1**			
**EMed-CP-wood#2-Y3**	1**	1**	1**	1	0.4		
**EMed-CP-wood#5-Y3**	1**	1**	1**	1	0.3	0.2	
**EMed-CP-wood#6-Y3**	1**	1**	1**	1	0.2	0.1	0.4
