# Supplementary material for: Temporal and Spatial Variations of Bacterial and Faunal Communities Associated with Deep-Sea Wood Falls
Source: PLoS One. 2017 Jan 25;12(1):e0169906. doi: 10.1371/journal.pone.0169906 (PMC5266260; doi:10.1371/journal.pone.0169906)
Supplement: S5 Table — Numbers represent percentages of the relative sequence abundance for each wood experiment. Analyses are based on the v6-extracted 454 MPTS dataset. (PDF) [file pone.0169906.s007.pdf]

EMed-CP-wood#1-Y1 (%)		EMed-CP-wood#1-Y3 (%)		EMed-CP-wood#2-Y1 (%)		EMed-CP-wood#2-Y3 (%)		EMed-CP-wood#5-Y1 (%)		EMed-CP-wood#5-Y3 (%)	
Alphaproteobacteria	19	Alphaproteobacteria	15	Alphaproteobacteria	27	Deltaproteobacteria	18	Alphaproteobacteria	24	Flavobacteriia	24
Deltaproteobacteria	16	Deltaproteobacteria	14	Gammaproteobacteria	18	Flavobacteriia	14	Flavobacteriia	14	Deltaproteobacteria	17
Clostridia	11	unclassified	13	Actinobacteria	10	Alphaproteobacteria	13	Gammaproteobacteria	11	Alphaproteobacteria	13
Gammaproteobacteria	9	Gammaproteobacteria	12	Flavobacteriia	9	unclassified	9	Deltaproteobacteria	8	Gammaproteobacteria	6
Flavobacteriia	5	Planctomycetacia	9	Sphingobacteriia	7	Gammaproteobacteria	8	Clostridia	5	SB-5	6
Bacteroidia	5	Epsilonproteobacteria	7	Planctomycetacia	4	SB-5	5	unclassified	4	Planctomycetacia	4
Spirochaetes	3	Actinobacteria	4	Deltaproteobacteria	4	Epsilonproteobacteria	4	Verrucomicrobiae	3	unclassified	4
unclassified	3	Flavobacteriia	4	Acidimicrobiia	4	Planctomycetacia	4	Sphingobacteriia	3	Sphingobacteriia	4
Deinococci	3	Acidimicrobiia	4	unclassified	4	Acidimicrobiia	3	Actinobacteria	3	Acidimicrobiia	3
Planctomycetacia	2	Clostridia	3	Cytophagia	3	Verrucomicrobiae	3	Acidimicrobiia	3	Epsilonproteobacteria	3

EMed-CP-wood#6-Y0 (%)		EMed-CP-wood#6-Y2 (%)		NorS-HMMV-wood#1-Y2 (%)		NorS-HMMV-wood#1-Y3 (%)	
Gammaproteobacteria	46	Deltaproteobacteria	22	Gammaproteobacteria	49	Alphaproteobacteria	30
Alphaproteobacteria	15	Flavobacteriia	16	Flavobacteriia	25	Flavobacteriia	24
Betaproteobacteria	10	Alphaproteobacteria	15	Alphaproteobacteria	21	Gammaproteobacteria	9
Actinobacteria	5	unclassified	8	Bacteroidia	1	Sphingobacteriia	8
Deltaproteobacteria	4	SB-5	7	unclassified	1	Deltaproteobacteria	7
Flavobacteriia	3	Planctomycetacia	4	Deltaproteobacteria	1	ARKICE-90	4
Epsilonproteobacteria	3	Gammaproteobacteria	4	Sphingobacteriia	1	Acidimicrobiia	4
Clostridia	2	Epsilonproteobacteria	4	Clostridia	0	Planctomycetacia	3
Acidobacteria	1	Acidimicrobiia	3	Mollicutes	0	Cytophagia	2
Dictyoglomia	1	Actinobacteria	2	Verrucomicrobiae	0	unclassified	2
