# Supplementary material for: Temporal and Spatial Variations of Bacterial and Faunal Communities Associated with Deep-Sea Wood Falls
Source: PLoS One. 2017 Jan 25;12(1):e0169906. doi: 10.1371/journal.pone.0169906 (PMC5266260; doi:10.1371/journal.pone.0169906)
Supplement: S6 Table — Relative sequence abundance, given as percentages, is shown in parenthesis. (PDF) [file pone.0169906.s008.pdf]

EMed-CP-woods-Y1	EMed-CP-woods-Y3	EMed-CP-wood#6-Y0	EMed-CP-wood#6-Y2
Tenacibaculum (8)	Pir4 lineage (8)	Pseudoalteromonas (22)	Pir4 lineage (10)
Demequina (7)	SEEP-SRB4 (8)	Vibrio (6)	Desulfobacula (8)
Leisingera (3)	Sulfurovum (7)	Moritella (6)	SEEP-SRB4 (8)
Blastopirellula (3)	Desulfobulbus (4)	Burkholderia (5)	Demequina (6)
Spirochaeta (2)	Desulfobacula (3)	Shewanella (3)	Desulfobulbus (5)
Conchiformibius (2)	Demequina (2)	Pseudomonas (3)	Sulfurimonas (5)
Marinifilum (2)	Leisingera (2)	Sulfurimonas (3)	Sulfurovum (5)
Maribacter (2)	Sulfurimonas (2)	Sphingomonas (3)	Desulforhopalus (4)
Prolixibacter (1)	Haloferula (2)	Marinospirillum (2)	Leisingera (3)
Desulfobacula (1)	Desulforhopalus (2)	Tenacibaculum (2)	Chthoniobacter (3)
Deinococcus (1)	Pacificibacter (2)	Propionibacterium (1)	Pacificibacter (2)
Leadbetterella (1)	Acidiferrobacter (1)	Dictyoglomus (1)	Reichenbachiella (2)
Haloferula (1)	Rhodobium (1)	Luteibacter (1)	Spirochaeta (2)
Roseobacter clade NAC11-7 lineage (1)	Spirochaeta (1)	Ralstonia (1)	Ulvibacter (2)
Pir4 lineage (1)	Ekhidna (1)	Erwinia (1)	Maribacter (1)
Roseobacter clade OCT lineage (1)	endosymbionts (1)	Frigoribacterium (1)	Caldithrix <1
Coxiella (1)	Reichenbachiella (1)	Methylobacterium <1	Antarctobacter <1
Reichenbachiella (1)	Leadbetterella (1)	Demequina <1	Peredibacter <1
Maritimibacter (1)	Desulfotalea (1)	Massilia <1	Ekhidna <1
Sulfurimonas (1)	Ralstonia	Pelomonas <1	Desulfotalea <1

NorS-HMMV-wood#1-Y2	NorS-HMMV-wood#1-Y3
Psychroserpens (30)	Pseudahrensia (24)
Marine Methylotrophic Group 3 (22)	Maribacter (13)
Simiduia (18)	Tenacibaculum (11)
Tenacibaculum (7)	Arenicella (7)
Pseudoalteromonas (5)	Formosa (5)
Marinifilum (4)	Bythopirellula (4)
Pricia (3)	Granulosicoccus (4)
Pacificibacter (3)	CL500-29 marine group (2)
Desulfopila (2)	Leisingera (2)
Pseudahrensia (1)	Leadbetterella (2)
Arenicella <1	Demequina (2)
Crocinitomix <1	Marine Methylotrophic Group 3 (2)
Maribacter <1	Ahrensia (2)
Mycoplasma <1	Algoriphagus (2)
Leisingera <1	Phycisphaera (1)
Colwellia <1	Roseobacter clade NAC11-7 lineage (1)
Desulfotalea <1	Blastopirellula (1)
Sulfitobacter <1	Persicirhabdus <1
Persicirhabdus <1	Subsaxibacter <1
Streptococcus <1	CL500-3 <1

EMed-CP-At-wood_0-2cm-Y1	EMed-CP-At-wood_0-2cm-Y3	EMed-CP-At-wood_5-7cm-Y3
Coxiella (12)	Pir4 lineage (11)	SEEP-SRB1 (37)
Methylobacterium (5)	Desulfarculus (10)	Desulfocapsa (13)
Ralstonia (4)	Coxiella (9)	Coxiella (8)
SEEP-SRB2 (2)	Christensenella (9)	Desulfobulbus (8)
Sphingomonas (2)	Desulfobacterium (8)	Acidiferrobacter (6)
Desulfobacula (2)	Desulfobacula (7)	Litoricola (3)
Propionibacterium (2)	Desulfobulbus (4)	Propionibacterium (3)
Legionella (2)	Sulfurovum (4)	Escherichia-Shigella (3)
Reichenbachiella (2)	SEEP-SRB4 (3)	Desulfopila (2)
Pseudomonas (1)	Sva0081 sediment group (3)	Mariprofundus (1)
Desulfobulbus (1)	Legionella (2)	Chryseobacterium (1)
Blastopirellula (1)	Caldithrix (2)	Bradyrhizobium (1)
Pir4 lineage (1)	Rhodobium (2)	Ralstonia (1)
SEEP-SRB1 (1)	Spirochaeta (2)	Sulfurovum (1)
Altererythrobacter (1)	Desulfopila (1)	Pir4 lineage (1)
Acinetobacter (1)	SEEP-SRB1 (1)	Altererythrobacter (1)
Streptococcus (1)	Sulfurimonas (1)	Caldithrix (1)
Spirochaeta (1)	Escherichia-Shigella (1)	Legionella (1)
Prolixibacter (1)	endosymbionts (1)	Pelomonas (1)
Acidiferrobacter (1)	Desulforhopalus (1)	Caldicellulosiruptor (1)
