# Supplementary material for: Temporal and Spatial Variations of Bacterial and Faunal Communities Associated with Deep-Sea Wood Falls
Source: PLoS One. 2017 Jan 25;12(1):e0169906. doi: 10.1371/journal.pone.0169906 (PMC5266260; doi:10.1371/journal.pone.0169906)
Supplement: S7 Table — Testing for significant differences in the bacterial community structures between wood experiments deployed for 3 years at spatially distant and close seeps. Asterisk denotes the level of statistical significance (p = 0.001). Analyses were based on the ARISA dataset. Similarity between wood bacterial community structures significantly decreased with increasing geographic distance between the wood experiments (454 data Mantel r = 0.6, p = 0.01, ARISA data Mantel r = 0.5, p = 0.01). (PDF) [file pone.0169906.s009.pdf]

	**EMed-CP-wood#1-Y3**	**EMed-CP-wood#2-Y3**	**EMed-CP-wood#3-Y3**	**EMed-CP-wood#4-Y3**	**EMed-CP-wood#5-Y3**	**NorS-HMMV-wood#1-Y3**
**EMed-CP-wood#1-Y3**						
**EMed-CP-wood#2-Y3**	0.3					
**EMed-CP-wood#3-Y3**	0.4***	0.3***				
**EMed-CP-wood#4-Y3**	0.4***	0.2	0			
**EMed-CP-wood#5-Y3**	0	0	0.2	0.1		
**NorS-HMMV-wood#1-Y3**	0.5***	0.5***	0.5***	0.4***	0.6***	
