# Supplementary material for: Temporal and Spatial Variations of Bacterial and Faunal Communities Associated with Deep-Sea Wood Falls
Source: PLoS One. 2017 Jan 25;12(1):e0169906. doi: 10.1371/journal.pone.0169906 (PMC5266260; doi:10.1371/journal.pone.0169906)
Supplement: S8 Table — Testing for significant differences in the bacterial community structures between wood-associated and sediment (wood-impacted “At wood” and reference “Away wood”) samples at (a) EMed-CP-wood#1 and (b) EMed-CP-wood#5 experiments immersed for 1y and 3y. ***p < 0.001, **p < 0.01, p < 0.05 after Bonferroni correction; (*) only significant without Bonferroni correction. Analyses were based on the ARISA dataset. (PDF) [file pone.0169906.s010.pdf]

a)	EMed-wood#1-Y1	EMed-Away_wood#1-Y1	EMed-At_wood#1-Y1	EMed-wood#1-Y3	EMed-At_wood#1-Y3
**EMed-Away_wood#1-Y1**	1**				
**EMed-At_wood#1-Y1**	0.91**	0.48			
**EMed-wood#1-Y3**	1**	0.98**	0.93**		
**EMed-At_wood#1-Y3**	0.96**	0.59	0.41	0.72**	
**EMed-Away_wood#1-Y3**	0.98**	0.72	0.78	0.74**	0.09

b)	EMed-wood#5-Y1	EMed-Away_wood#5-Y1	EMed-At_wood#5-Y1	EMed-wood#5-Y3	EMed-At_wood#5-Y3
**EMed-Away_wood#5-Y1**	1**				
**EMed-At_wood#5-Y1**	0.82**	0.33			
**EMed-wood#5-Y3**	0.99**	1(**)	0.65(**)		
**EMed-At_wood#5-Y3**	0.93**	1.00	0.63	0.80(**)	
**EMed-Away_wood#5-Y3**	0.99**	1.00	0.78	1(**)	0.48
