# Supplementary figures and images for: Temporal and Spatial Variations of Bacterial and Faunal Communities Associated with Deep-Sea Wood Falls
Source: PLoS One. 2017 Jan 25;12(1):e0169906. doi: 10.1371/journal.pone.0169906 (PMC5266260; doi:10.1371/journal.pone.0169906)

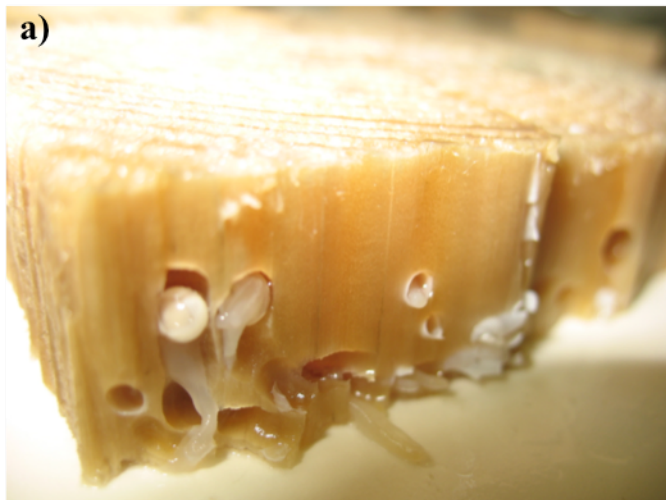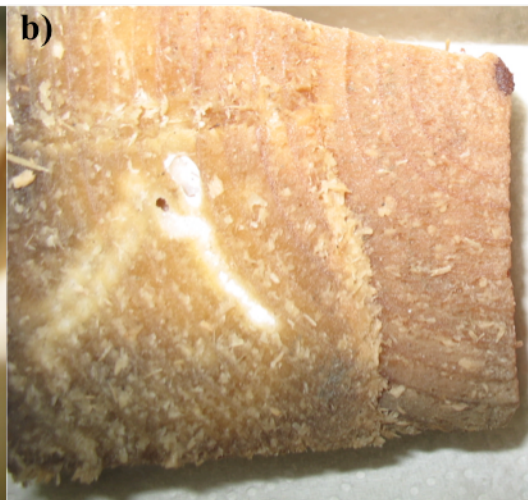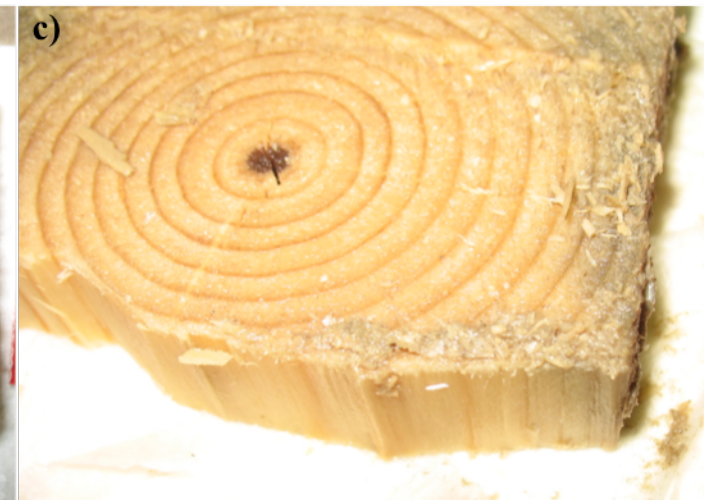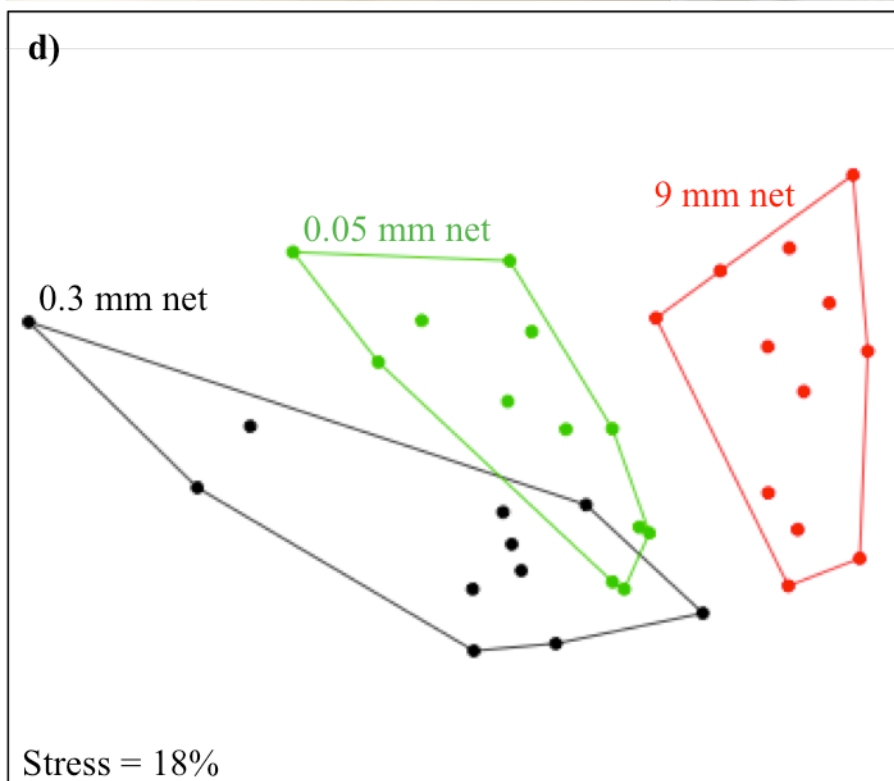

e)

|             | 9 mm net | 0.3 mm net | 0.05 mm net |
|-------------|----------|------------|-------------|
| 9 mm net    |          |            |             |
| 0.3 mm net  | 40       |            |             |
| 0.05 mm net | 40       | 36         |             |

f)

|             | 9 mm net | 0.3 mm net | 0.05mm net |
|-------------|----------|------------|------------|
| 9 mm net    |          | 0.0003     | 0.0003     |
| 0.3 mm net  | 0.6      |            | 0.02       |
| 0.05 mm net | 0.5      | 0.2        |            |

Supplement: S1 Fig — Photos showing wood-boring bivalves colonizing the wood tiles of the nets with 9 mm (a) and 0.3 mm (b) mesh size. (c) wood tiles in the net with the smallest mesh size (0.05 mm) were no populated by wood-boring bivalves. NMDS (d) and ANOSIM analyzes (f) revealing significant differences in the bacterial community structure between wood tiles of the different nets. (f) ANOSIM R values are shown in the lower and Bonferroni corrected p-values in the upper triangle. Percentages of shared OTUs between the wood tiles of the different nets are displayed in (e). (PDF) [file pone.0169906.s011.pdf]

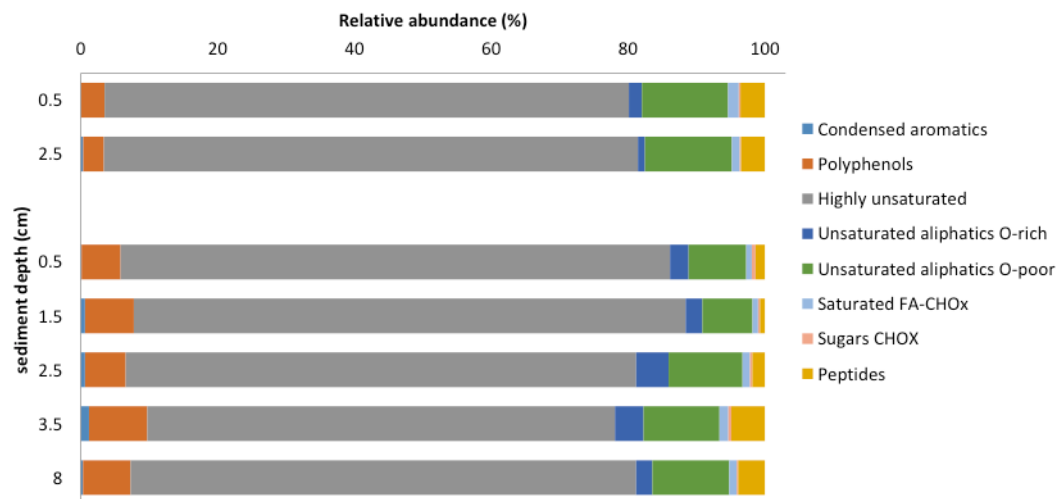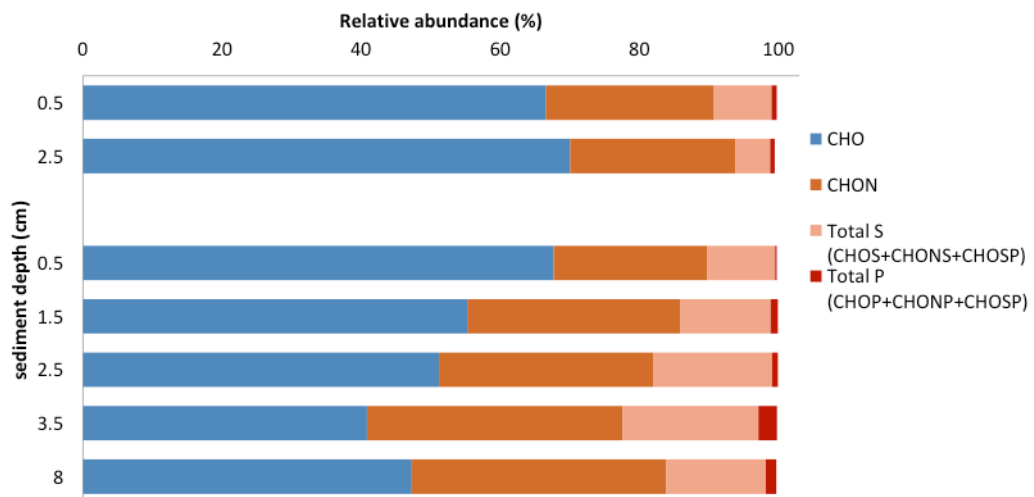

Supplement: S2 Fig — a) DOM molecular formulae observed in porewaters indicating the elemental composition in the different samples, and b) Major molecular groups at EMed-CP-wood#5 and its reference site. Bars on the right indicate samples that were directly compared, i.e. surface sediments at the reference site and the wood-chip sediment boundary layer at the wood experiment, where a 2–3 cm thick layer of wood chips had accumulated on top of the seafloor. (PDF) [file pone.0169906.s012.pdf]

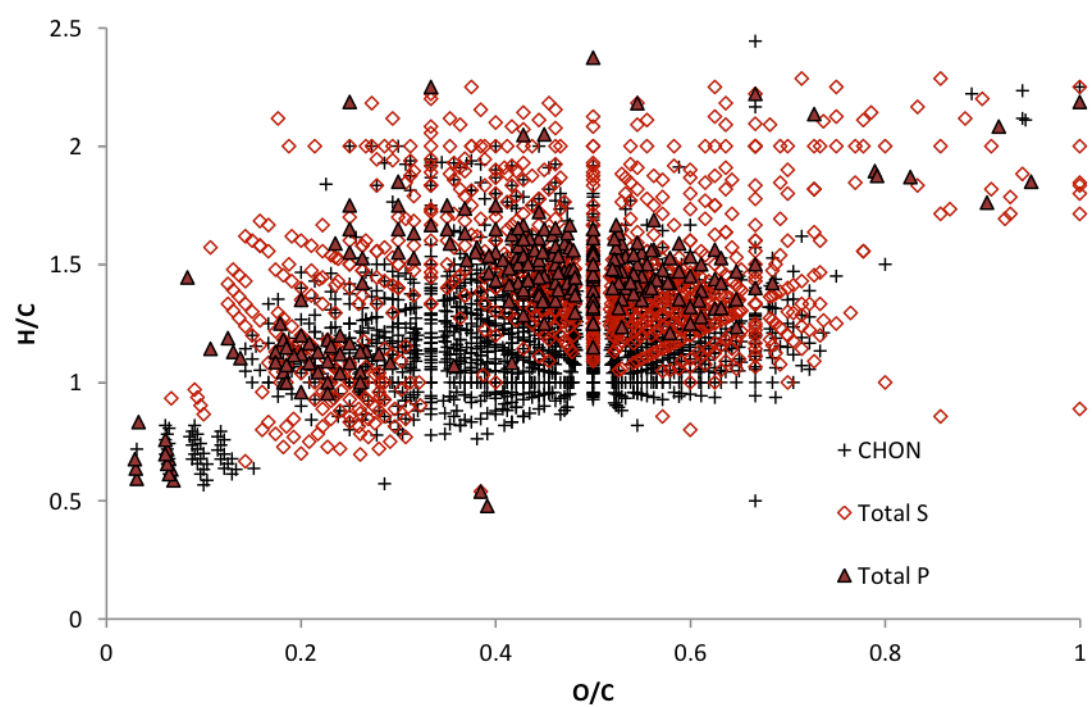

Supplement: S3 Fig — Unique formulae mainly belonged to groups containing CHON (1668 formulae), sulfur (958 formulae) and phosphorous (237 formulae); the latter two also include the combination of S and P with other elements such N. (PDF) [file pone.0169906.s013.pdf]

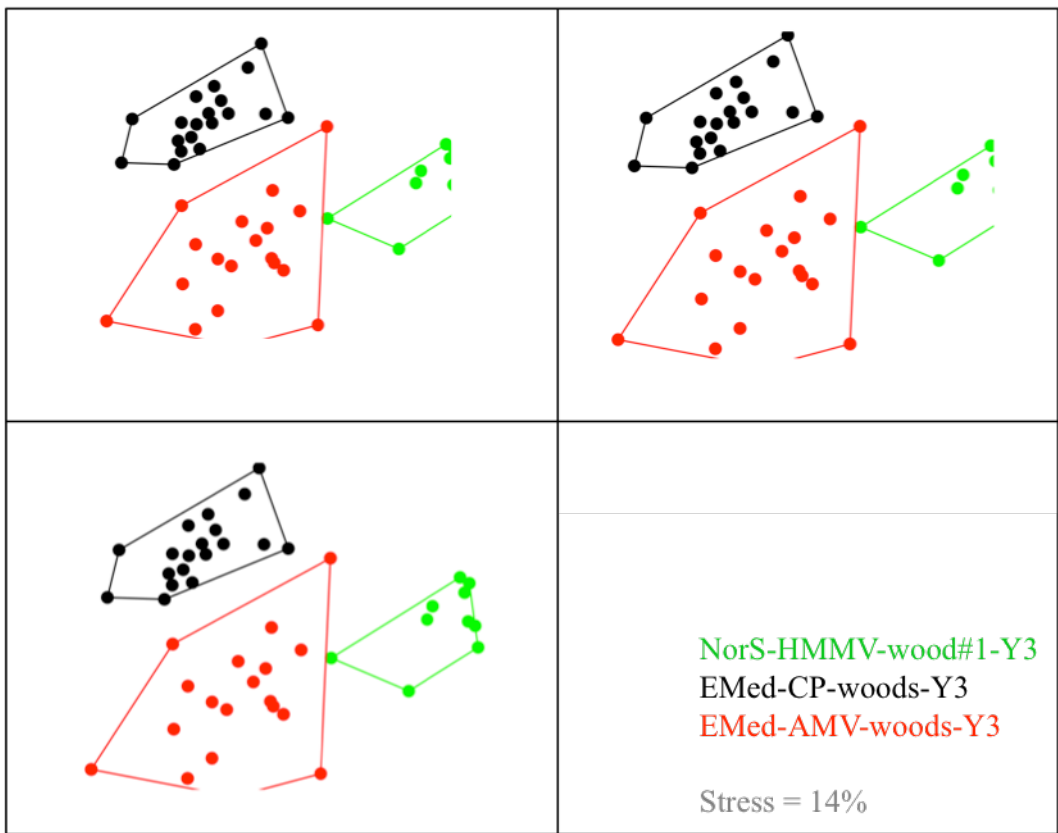

Supplement: S4 Fig — Samples are color coded according to location of wood experiments. Stress = 14%. (PDF) [file pone.0169906.s014.pdf]
